# Ancestral dichlorodiphenyltrichloroethane (DDT) exposure promotes epigenetic transgenerational inheritance of obesity

**DOI:** 10.1186/1741-7015-11-228

**Published:** 2013-10-23

**Authors:** Michael K Skinner, Mohan Manikkam, Rebecca Tracey, Carlos Guerrero-Bosagna, Muksitul Haque, Eric E Nilsson

**Affiliations:** 1 Center for Reproductive Biology, School of Biological Sciences, Washington State University, Pullman, WA 99164-4236, USA

**Keywords:** Environmental epigenetics, Metabolic syndrome, Obesity associated disease, Epimutations, Disease etiology, Maternal transmission

## Abstract

**Background:**

Ancestral environmental exposures to a variety of environmental factors and toxicants have been shown to promote the epigenetic transgenerational inheritance of adult onset disease. The present work examined the potential transgenerational actions of the insecticide dichlorodiphenyltrichloroethane (DDT) on obesity and associated disease.

**Methods:**

Outbred gestating female rats were transiently exposed to a vehicle control or DDT and the F1 generation offspring bred to generate the F2 generation and F2 generation bred to generate the F3 generation. The F1 and F3 generation control and DDT lineage rats were aged and various pathologies investigated. The F3 generation male sperm were collected to investigate methylation between the control and DDT lineage male sperm.

**Results:**

The F1 generation offspring (directly exposed as a fetus) derived from the F0 generation exposed gestating female rats were not found to develop obesity. The F1 generation DDT lineage animals did develop kidney disease, prostate disease, ovary disease and tumor development as adults. Interestingly, the F3 generation (great grand-offspring) had over 50% of males and females develop obesity. Several transgenerational diseases previously shown to be associated with metabolic syndrome and obesity were observed in the testis, ovary and kidney. The transgenerational transmission of disease was through both female (egg) and male (sperm) germlines. F3 generation sperm epimutations, differential DNA methylation regions (DMR), induced by DDT were identified. A number of the genes associated with the DMR have previously been shown to be associated with obesity.

**Conclusions:**

Observations indicate ancestral exposure to DDT can promote obesity and associated disease transgenerationally. The etiology of disease such as obesity may be in part due to environmentally induced epigenetic transgenerational inheritance.

## Background

A number of environmental factors such as toxicants and nutrition have been shown to promote the epigenetic transgenerational inheritance of adult onset disease and phenotypic variation [1-3]. Examples of environmental compounds include the fungicide vinclozolin [4-6], plasticizers bisphenol-A (BPA) and phthalates [7], dioxin [7-9], hydrocarbons [7,8], and pesticides [4,7,8]. Nutritional abnormalities such as caloric restriction and high-fat diets can also promote transgenerational phenotypes [10]. Epigenetic transgenerational inheritance involves the germline (sperm or egg) transgenerational transmission of epigenetic marks that influence physiological parameters and disease, in the absence of direct environmental exposures [1,3]. This phenomenon has been observed in plants [11], flies [12], worms [13], rodents [4], and humans [14]. Therefore, your ancestors? environmental exposures may influence your disease development, even though you have never had a direct exposure. Environmentally induced epigenetic transgenerational inheritance of disease appears to be a factor in disease etiology that needs to be considered and elucidated.

The present study was designed to examine the potential transgenerational actions of the most common historically used insecticide dichlorodiphenyltrichloroethane (DDT) [15,16]. DDT has been banned from the USA, but is used globally as an insecticide for control of vectors for malaria and visceral leishmaniasis. DDT is 1 of the 12 chemicals proposed for elimination by the 2001 Stockholm Convention of United Nations Environmental Program [17]. However, DDT use in Africa has increased since the Stockholm Convention due to the recent Gates Foundation Malaria Control Program [18]. The reported global use of DDT for disease vector control is 4,000 to 5,000 metric tons per year, with India being by far the largest consumer [19]. In 2006, the World Health Organization issued a position statement promoting the use of indoor residual spraying with DDT for malaria vector control. Although DDT is a low-cost antimalarial tool, the possible adverse human health and environmental effects of exposure must be carefully weighed against the benefits to malaria control [16]. Recent evidence indicates that indoor spraying causes high levels of human exposure to DDT [20]. The direct exposure toxic effects of DDT in humans have been reviewed [15] and include reproductive disease [21], neurological disease [22], developmental abnormalities [23], and cancer [24]. Studies have also shown DDT?s potential to cause birth defects in wildlife [25]. Exposure to DDT and its breakdown product dichlorodiphenyldichloroethylene (DDE) may be associated with adverse health outcomes such as diabetes and obesity in children [26,27].

The dramatic increase in obesity over the past 50 years has suggested environmental factors are important in the disease etiology. The prevalence of obesity has increased substantially since the mid-20th century with an accelerated rate of increase in the 1980s [28]. The US Centers for Disease Control in 2010 reported that 33% of adults in the US are obese and 17% of children between ages 2 to 19 are obese. Obesity has not only increased in the US, but also increased in virtually every country where detailed data are available [29]. The primary causal factor suggested is overnutrition [28], however, recent studies have suggested environmental toxicants [28] such as plastics [30,31], hydrocarbons [32], and tributyltin [33] can promote obesity in rodents. Although overnutrition and reduced physical activity are critical elements of the disease, other contributing factors include maternal age, endocrine disruptors, sleep deprivation, pharmaceutical introgenesis, ambient temperature, and intrauterine and intergenerational effects [28]. All these contributing factors have been shown to be involved in obesity, but the underlying molecular mechanisms are unclear. Genetic abnormalities have been identified in a number of the genes associated with obesity [34,35], however, no significant genome-wide associations have been shown to correlate with the majority of obese individuals [35]. In addition, no known genetic mechanism could explain the rapid increase in the incidence of obesity in the last 30 years. Clearly, genetics will be a critical aspect of any disease, including obesity, but it simply cannot explain many of the elements of the disease etiology. An alternate consideration is the role of environmental epigenetics in obesity [28] and in the developmental origins of disease [36]. The present study further investigates the role of epigenetics in the etiology of obesity.

Obesity is now known to be associated with a number of different clinical conditions in a complex disease trait known as metabolic syndrome [37]. Although a number of diseases have associations, the functional link and correlation remains to be elucidated. Predominant associated conditions are insulin resistance [38] and polycystic ovarian disease [39]. Other obesity-associated conditions include type 2 diabetes [40], non-alcoholic fatty liver disease [41], obstructive sleep apnea [42], kidney/renal disease [43] and testis disease and male infertility [44]. The scientific literature suggests over 50% of females with polycystic ovarian disease are obese [45,46]. Therefore, polycystic ovarian disease and several other diseases that are associated with obesity are also investigated in the present study.

Although the direct exposure toxicity of DDT is documented [47], no previous transgenerational studies involving DDT exposure have been reported. The present study tests the hypothesis that DDT promotes the epigenetic transgenerational inheritance of obesity and associated disease. In the event DDT ancestral exposures promote obesity in subsequent generations, in the absence of any direct exposures, the biohazards of DDT are significantly greater than anticipated. It may be that ancestral exposures to environmental toxicants such as DDT have a significant role in the etiology of the obesity observed in the current human population. Interestingly, the F3 generation of the majority of the gestating women exposed in the 1950s in the USA are adults today. The elucidation of the epigenetic biomarkers and molecular mechanisms involved in this environmentally induced epigenetic transgenerational inheritance is anticipated to lead to new diagnostics and therapeutics for obesity and associated diseases.

## Methods

### Animal studies and breeding

Female and male rats of an outbred strain Hsd:Sprague Dawley??SD?? Harlan (Indianapolis, IN) at about 70 and 100 days of age were fed *ad libitum* with a standard rat diet and ad lib tap water for drinking. To obtain time-pregnant females, the female rats in proestrus were pair mated with male rats. The sperm-positive (day 0) rats were monitored for diestrus and body weight. On days 8 to 14 of gestation [48], the females were administered daily intraperitoneal injections of DDT (either 50 or 25 mg/kg BW/day) or dimethyl sulfoxide (vehicle). The p,p?-DDT was obtained from Sigma (St Louis, MO, USA) (no. PS699) and was injected in a 20 ?l dimethylsulfoxide (DMSO)/oil vehicle as previously described [7]. Treatment lineages are designated ?control?, ?DDT? or ?lower? dose DDT lineages. This is not meant to represent a ?low? dose analysis. The gestating female rats treated were designated as the F0 generation. The offspring of the F0 generation rats were the F1 generation. Non-littermate females and males aged 70 to 90 days from F1 generation of control, DDT or low dose DDT lineages were bred to obtain F2 generation offspring. The F2 generation rats were bred to obtain F3 generation offspring. Outcross F4 generation offspring (n?=?8 litters per lineage) were obtained by breeding the F3 generation males from control and low dose DDT lineages with wild type females. Reverse outcross F4 generation progeny (n?=?8 litters per lineage) were obtained by breeding the F3 generation females from control and low dose DDT lineages with wild type males. The outcross and the reverse outcross individuals were maintained until 10 months of age and then euthanized for tissue collection and disease evaluation. The F1 to F4 generation offspring were not themselves treated directly with DDT. The control and DDT lineages were housed in the same room and racks with lighting, food and water as previously described [1,5,7]. All experimental protocols for the procedures with rats were preapproved by the Washington State University Animal Care and Use Committee (IACUC approval no. 02568?029).

### Tissue harvest and histology processing

Rats at 10 to 12 months of age were euthanized by CO_2_ inhalation for tissue harvest. Body and organ weights were measured at dissection time. No significant changes in body weight were observed within this 2-month period and statistical analysis did not identify this as a confounder in the analysis. Testis, epididymis, prostate, seminal vesicle, ovaries, uterus and kidney were fixed in Bouin?s solution (Sigma) and 70% ethanol, then processed for paraffin embedding by standard procedures for histopathology examination. Tissue sections of 5 ?m were made and were either unstained and used for terminal deoxynucleotidyl transferase-mediated dUTP nick-end labeling (TUNEL) analysis or stained with hematoxylin and eosin (H&E) stain and examined for histopathology. Blood samples were collected at the time of dissection, allowed to clot, centrifuged and serum samples stored for steroid hormone assays.

### Histopathology examination and disease classification

Obesity was assessed with an increase in body weight and marked abdominal adiposity. The obesity classification has been defined as these abnormalities and the presence of associated pathologies [28,36,49-51]. Testis histopathology criteria included the presence of a vacuole, azoospermic atretic seminiferous tubule and ?other? abnormalities including sloughed spermatogenic cells in center of the tubule and a lack of a tubule lumen. Testis sections were examined by TUNEL assay (*in situ* cell death detection kit, Fluorescein, Roche Diagnostics, Mannheim, Germany). Prostate histopathology criteria included the presence of vacuoles, atrophic epithelial layer of ducts and hyperplasia of prostatic duct epithelium as previously described [52,53]. No prostatic intraepithelial neoplasia (PIN) lesions were observed in the prostates. Kidney histopathology criteria included reduced size of glomerulus, thickened Bowman?s capsule and the presence of proteinaceous fluid-filled cysts. A cut-off was established to declare a tissue ?diseased? based on the mean number of histopathological abnormalities plus 2 standard deviations from the mean of control tissues by each of the three individual observers. This number was used to classify rats into those with and without testis, prostate or kidney disease in each lineage. A rat tissue section was finally declared ?diseased? only when at least two of the three observers marked the same tissue section ?diseased?. The proportion of rats with obesity or tumor development was obtained by accounting those that had these conditions out of all the animals evaluated.

Ovary sections were stained with H&E stain and three stained sections (150 ?m apart) through the central portion of the ovary with the largest cross section were evaluated. Ovary sections were assessed for two diseases, primordial follicle loss and polycystic ovary disease. Primordial follicle loss was determined by counting the number of primordial follicles per ovary section and averaging across three sections. An animal was scored as having primordial follicle loss if the primordial follicle number was less than that of the control mean minus 2 standard deviations. Primordial follicles had an oocyte surrounded by a single layer of either squamous or both squamous and cuboidal granulosa cells [8,54]. Follicles had to be non-atretic and showing an oocyte nucleus in order to be counted. Polycystic ovary was determined by microscopically counting the number of small cystic structures per section averaged across three sections. A polycystic ovary was defined as having a number of small cysts that was more than the control mean plus 2 standard deviations. Cysts were defined as fluid-filled structures of a specified size that were not filled with red blood cells and which were not follicular antra. A single layer of cells may line cysts. Small cysts were 50 to 250 ?m in diameter measured from the inner cellular boundary across the longest axis. Percentages of females with primordial follicle loss or polycystic ovarian disease were computed.

### Overall disease incidence

A table of the incidence of individual diseases in rats from each lineage was created and the proportion of individual disease, total disease and multiple disease incidences was computed. For the individual diseases, only those rats that showed a presence of disease (plus) or absence of disease (minus) are included in the computation. For the total diseases, a column with total number of diseases for each rat was created and the number of plus signs were added up for each of the rats and the proportion was computed as the number of rats with total disease out of all the listed rats. For the multiple diseases, the proportion was computed as the number of rats with multiple diseases out of all the listed rats.

### Epididymal sperm collection and DNA isolation and methylated DNA immunoprecipitation

The epididymis was dissected free of connective tissue, a small cut made to the cauda and placed in 5 ml of F12 culture medium containing 0.1% bovine serum albumin for 10 minutes at 37?C and then kept at 4?C to immobilize the sperm. The epididymal tissue was minced and the released sperm centrifuged at 13,000 *g* and stored in fresh nuclear isolation medium (NIM) buffer at ?20?C until processed further. Sperm heads were separated from tails through sonication following previously described protocol (without protease inhibitors) [55] and then purified using a series of washes and centrifugations [56] from a total of nine F3 generation rats per lineage (control or DDT) that were 120 days of age. DNA extraction on the purified sperm heads was performed as described [57]. Equal concentrations of DNA from three individual sperm samples were used to produce three DNA pools per lineage and employed for chromatin immunoprecipitation of methylated DNA fragments (MeDIP). MeDIP was performed as previously described [7,57].

### MeDIP-chip analysis

The comparative MeDIP-chip was performed with Roche Nimblegen?s Rat DNA Methylation 3???720 K CpG Island Plus RefSeq Promoter Array which contains 3 identical subarrays, with 720,000 probes per subarray, scanning a total of 15,287 promoters (3,880 bp upstream and 970 bp downstream from transcription start site). Probe sizes range from 50 to 75 bp in length with the median probe spacing of 100 bp. Three different comparative (MeDIP vs MeDIP) hybridization experiments were performed (three subarrays) for DDT lineage versus control, with each subarray encompassing DNA samples from six animals (three each from DDT and control). MeDIP DNA samples from experimental lineages were labeled with Cy3 and MeDIP DNA samples from the control lineage were labeled with Cy5. Selected differential DNA methylation regions (DMR) identified with the MeDIP-chip analysis were confirmed with a MeDIP-quantitative polymerase chain reaction (QPCR) analysis involving real-time PCR analysis of the MeDIP samples as previously described [31,32].

### Bioinformatic and statistical analyses of MeDIP-chip data

The bioinformatic analysis was performed as previously described [7,57]. The statistical analysis was performed in pairs of comparative immunoprecipitation hybridizations between DDT (D) and controls (C) (for example, D1-C1 and D2-C2, D1-C1 and D3-C3, D2-C2 and D3-C3). In order to assure the reproducibility of the candidates obtained, only the candidates showing significant changes in all of the single paired comparisons (intersection) were chosen as a having a significant change in DNA methylation between DDT lineage and control lineage. This is a very stringent approach to select for changes, since it only considers repeated changes in all paired analysis. The statistically significant differential DNA methylated regions were identified and *P* value associated with each region presented. Each region of interest was then annotated for gene and CpG content. This list was further reduced to those regions with an average intensity value exceeding 9.5 (log scale) and a CpG density ?1 CpG/100 bp.

### Statistical analysis of rat organ and disease data

Individual animals from different litters were used for analysis and n values presented for all experiments. For statistical analysis, all the continuous data on body and organ weights and apoptosis were used as input in the program GraphPad Prism 5 statistical analysis program and t tests were used to determine if the data from the DDT lineages differ from those of control lineages. For the number of rats with or without disease, logistic regression analysis was used to analyze the data (control or DDT and diseased or unaffected). A simple logistic regression was performed using an online calculator tool (http://vassarstats.net/logreg1.html). The binary outcome variable was diseased/not diseased (for example, obese/non-obese). The predictor variable was treatment (control vs DDT and control vs LD DDT) and each was performed separately for the analysis. Each treatment group was only compared to its own control using the numbers of affected/non-affected individuals evaluated for each treatment group. All treatment differences were considered significant if the *P* value was less than 0.05.

## Results

### Transgenerational obesity and associated disease analysis

The transgenerational actions of control (vehicle DMSO), DDT (50 mg/kg body weight) and a lower dose (LD) DDT (25 mg/kg BW) administered female rats (F0 generation) during days 8 to 14 of gestation were investigated. The doses of DDT used are anticipated environmental exposures [22,58]. The F1 (direct exposure) and F3 (transgenerational) generation rats of control, DDT and lower dose DDT lineages were aged to 1 year and euthanized for analysis. The testis, prostate, kidney, ovary and uterus were collected and examined for histopathologies. To assess if there was any direct fetal exposure toxicity to DDT the F1 generation litter size, sex ratio, body weights and organ weights were measured (Additional file 1: Table S1A). No effect was observed on litter size or sex ratio (*P* >0.05). The body weights of the F1 generation DDT and LD DDT were slightly reduced, while several organ weights were slightly increased. Therefore, no overt toxicity to DDT was observed in the direct *in utero* exposed F1 generation lineages.

The incidence of obesity in DDT and LD DDT lineages are presented in Figure?1a,b. The obesity was determined using an increase in body weight and abdominal adiposity (fat deposition) and presence of associated disease. The body weights of the non-obese (506.6???8.2 g, male; 278.4???2.7 g, female) compared to the obese (515???5.8 g, male; 297.1???4.2 g, female) DDT and LD DDT lineages indicated a statistically significant increase in body weight in the DDT and LD DDT lineage obese animals (*P* <0.05). Although the mean body weight for all DDT and LD DDT lineage individuals was not increased (Additional file 1: Table S1), all the obese animals did have an increase in body weight. The magnitude of the statistically significant weight gain was not large, but we did observe larger weight gain as the animals aged, as previously described [59]. No weight gain effect was observed on younger 120-day-old animals, indicating the obesity weight gain was an adult onset condition. Analysis of the abdominal adiposity for a non-obese (Figure?1c) compared to an obese (Figure?1d) animal demonstrated a dramatic increase in abdominal fat deposition (adiposity) on nearly all organs in obese animals. An increased body weight and abdominal adiposity was observed in all the obese animals identified. Interestingly, no increase in the incidence of obesity was observed in the F1 generation DDT or LD DDT lineages (Figure?1). In contrast, the F3 generation LD DDT lineage females and males had 50% of the animals develop obesity. In the F3 generation DDT lineage males 75% of the animals developed obesity. Although the DDT and LD DDT F1 generation animals did not have an altered incidence of obesity, the ancestral DDT and LD DDT exposures were found to promote transgenerational (F3 generation) obesity in the majority of males and females. Therefore, DDT was found to promote the transgenerational inheritance of obesity and as discussed below the molecular mechanisms of F1 and F3 generation disease are distinct.

**Figure 1:**
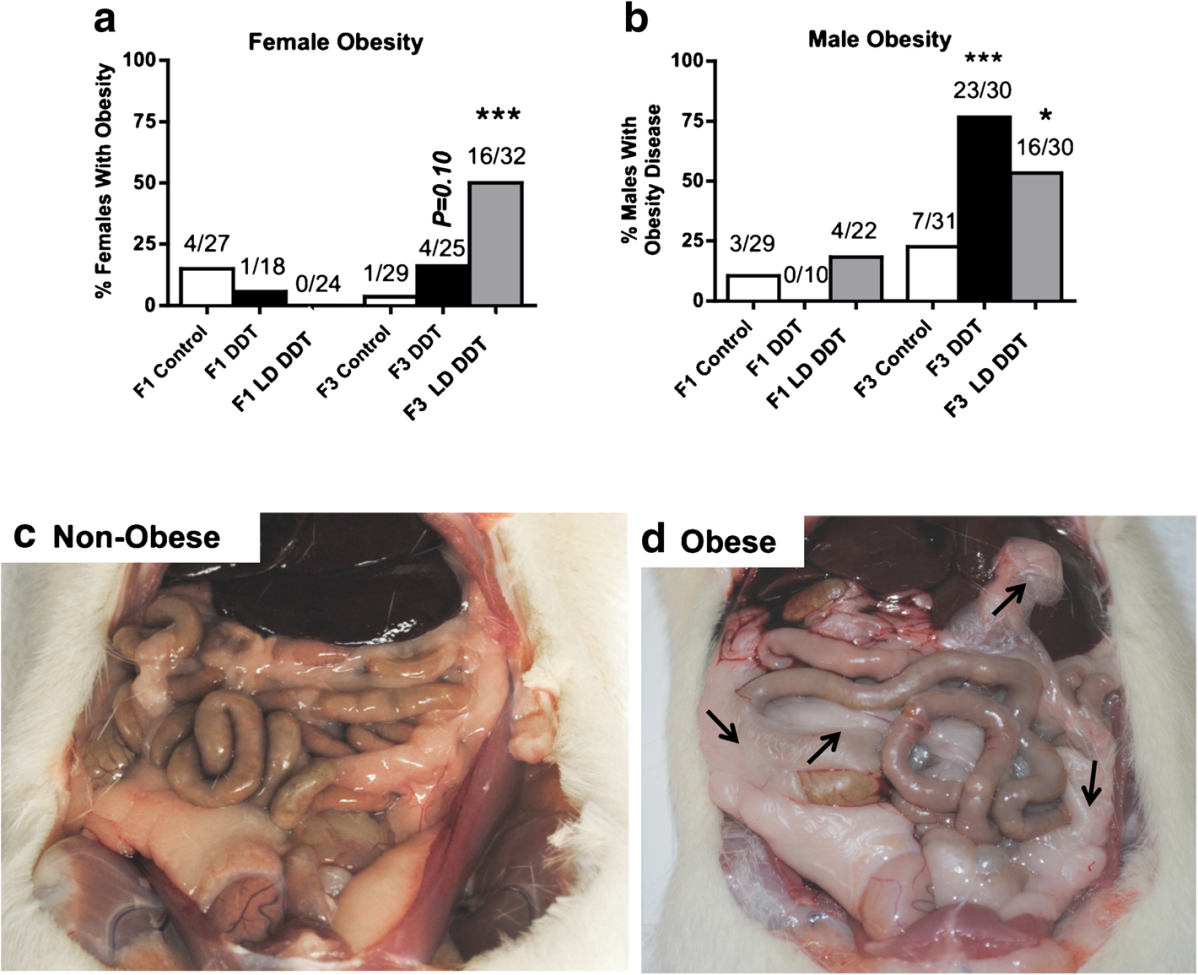
**Transgenerational obesity.** Percentages of females **(a)** or males **(b)** with obesity in the F1 and F3 generations. The dichlorodiphenyltrichloroethane (DDT) or lower dose (LD) DDT is indicated. The number of diseased rats/total number of rats (n value) is shown above the respective bar graphs. A logistic regression analysis was performed and *P* values indicate a significant difference from controls (*P <0.05; **P <0.01; ***P <0.001). The representative abdominal adiposity is shown for non-obese **(c)** and obese **(d)** rats, with the pink fat tissue deposition indicated (arrows) around the liver, intestines, subcutaneous and epididymis in the obese animal.

Since obesity is a component of a complex disease trait (Figure?2), a number of the other obesity-associated diseases (for example, testis disease, polycystic ovarian disease, and kidney disease) were investigated. The causal correlation of these diseases remains to be determined, but the presence of associated disease is presented. The incidence of testis disease in DDT and LD DDT lineages is presented in Figure?3a. Testis disease was characterized by the presence of histopathology including azoospermia, atretic seminiferous tubules, presence of vacuoles in basal regions of seminiferous tubules, sloughed germ cells in the lumen of seminiferous tubules, and lack of seminiferous tubule lumen (Additional file 2: Figure S1A). DDT exposure had no influence on testis disease in the F1 generation males. Testis disease incidence increased significantly in the F3 generation DDT lineage with 47% of the males affected (Figure?3a). Further analysis of the testis examined the number of apoptotic spermatogenic cells in the testis and epididymal sperm counts (Figure?3b,c). Spermatogenic cell apoptosis was decreased in the F1 generation DDT and LD DDT lineages and increased in the F3 generation lineages compared to control. Sperm counts were decreased in the F3 generation DDT lineage males. Therefore, DDT was found to promote obesity-associated transgenerational testis disease.

**Figure 2:**
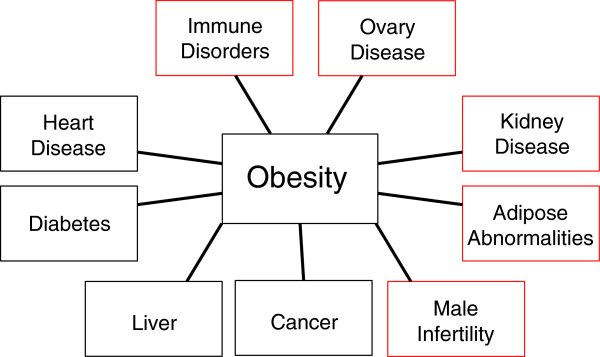
**Obesity and associated disease.** The major diseases previously shown to be associated with obesity are indicated with those identified in the present study shown with a red box.

**Figure 3:**
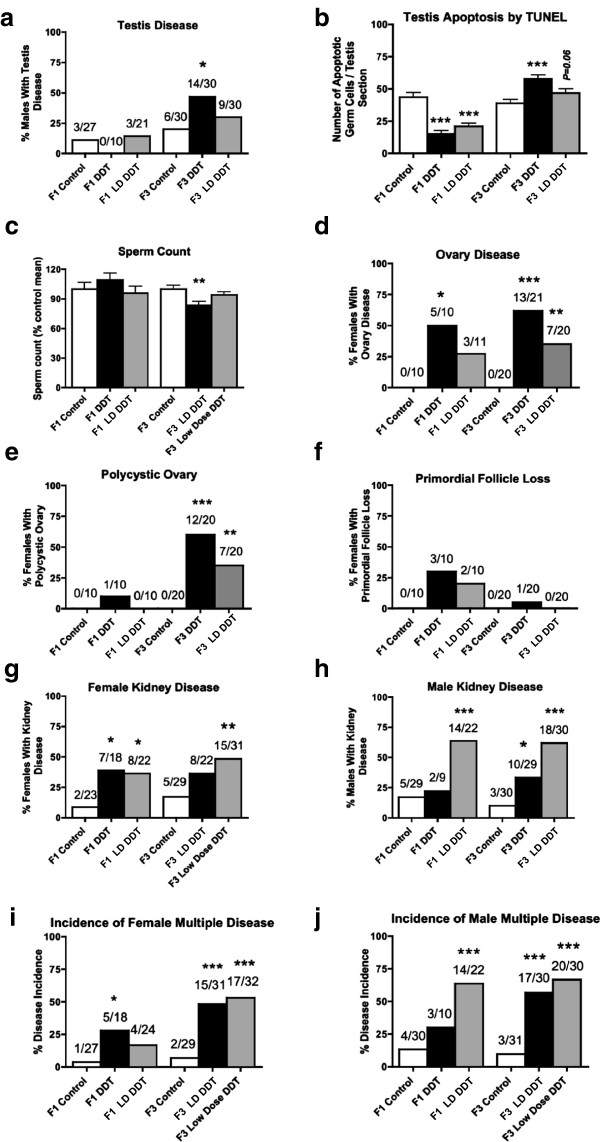
**Transgenerational obesity and associated disease.** Percentages of the F1 and F3 generation disease/abnormalities from control (open bars), dichlorodiphenyltrichloroethane (DDT) (black bars) and lower dose (LD) DDT lineages. Testis disease **(a)**, spermatogenic cell apoptosis **(b)** and sperm counts **(c)**, ovarian disease **(d)**, polycystic ovarian disease **(e)** and primordial follicle pool loss **(f)** are presented. Percentages of females **(g)** and males **(h)** with kidney diseases and percentages of females (I) and males (J) with incidence of multiple disease. The number of diseased rats/total number of rats (n value) is shown above the respective bar graphs. Those showing numbers above the bars were analyzed with a logistic regression analysis and those with a mean???SEM indicated were analyzed with a *t* test with the *P* value indicated (*P <0.05; **P <0.01; ***P <0.001) (Additional file 4: Table S2 and Additional file 5: Table S3).

The incidence of ovarian disease in DDT and LD DDT lineages is presented in Figure?3d. An increase in ovarian disease was observed in both the F1 and F3 generation DDT lineages. The primary ovarian disease detected was the development of polycystic ovaries with an increase in the number of small and large cysts as previously described [60] (Figure?3e). In contrast to previous environmental toxicants examined [60], no effect on primordial follicle numbers were detected in the DDT or LD DDT lineages (Figure?3f). The increase in ovarian disease in the F3 generation DDT and LD DDT lineages observed was primarily characterized by the development of ovarian cysts. Analysis of estradiol levels in the proestrous or diestrous F3 generation females revealed this was not changed in the DDT or LD DDT lineages (Additional file 3: Figure S2C,D). Therefore, DDT and LD DDT were found to promote the obesity-associated transgenerational polycystic ovarian disease.

The incidence of kidney disease in DDT and LD DDT lineages is presented in Figure?3g and 3h. Kidney disease was characterized by the presence of an increased number of proteinaceous fluid filled cysts, reduction in size of glomeruli and thickening of Bowman?s capsules (Additional file 2: Figure S1B). There was an increase in female kidney disease in F1 and F3 generation LD DDT lineage, but only in the F1 generation in the DDT lineage (Figure?3g). The males showed a dramatic increase with over 60% of the F1 and F3 generation animals in the LD DDT lineage affected (Figure?3h). Therefore, DDT was found to promote the obesity-associated transgenerational kidney disease in both males and females.

Additional potential obesity-associated disease and abnormalities (Figure?2) investigated were prostate disease [61], pubertal abnormalities [62], tumor development [63] and immune abnormalities [64]. Prostate disease was examined in the F1 and F3 generation males (Additional file 2: Figure S1 and Table S2A). Prostate disease was characterized by atrophic prostate duct epithelium and hyperplasia (Additional file 2: Figure S1A) as previously described [52]. The incidence of prostate disease increased in F1 generation males of DDT and LD DDT lineages, but no effect was observed in the F3 generation males. Therefore, no transgenerational prostate disease was observed. In addition, no influence on F3 generation male testosterone levels was observed (Additional file 3: Figure S2E). Analysis of the onset of puberty, as previously described [7], was found not to be altered in either females (Additional file 3: Figure S2F) or males (Additional file 3: Figure S2G) in the F1 or F3 generations. The incidence of tumor development was monitored and the primary tumors observed were mammary tumors, as previously described [5]. No altered tumor development was observed in the F1 or F3 generation females (Additional file 3: Figure S2H). An increase in tumor development was observed in the F1 generation DDT lineage males, but no effect was observed in the F3 generation lineages (Additional file 3: Figure S2I). Therefore, no transgenerational influence was observed for prostate disease, pubertal abnormalities or tumor development. Analysis of the uterus identified a significant increase in uterine infection in the F3 generation DDT lineage females, with over 70% of the animals affected (Additional file 3: Figure S2B). Uterine infection was determined by the enlargement of uterus, accumulation of foul-smelling dark discolored purulent material and presence of inflammation within the uterine horns. Therefore, potential transgenerational immune abnormalities were detected in the females.

The incidence of multiple disease and abnormalities (?2) per rat was determined (Figure?3i,j). The incidence of diseases in individual rats from control, DDT and LD DDT lineages are presented in Additional file 4: Table S2 for F1 generation females (S2A) and males (S2B), and in Additional file 5: Table S3 for F3 generation females (S3A) and males (S3B). The incidence of multiple diseases in F1 generation females increased in the DDT lineage, while the incidence in the F3 generation females increased in approximately 50% of the animals in the DDT and LD DDT lineages (Figure?3i). The incidence of multiple disease in the F1 generation males increased in the LD DDT lineage, while the incidence in the F3 generation males increased to affect approximately 60% of the animals in the DDT and LD DDT lineages (Figure?3j). Therefore, exposure of F0 generation females to two different doses of DDT increased the incidence of obesity and associated multiple diseases in the F1 and F3 generation male and female progeny.

### Parental germline transmission of DDT-induced transgenerational obesity and associated disease

An experiment was designed to determine if the epigenetic transgenerational inheritance of adult onset obesity and associated disease is transmitted through the male (sperm) and/or female (egg) germline. The F3 generation control and LD DDT lineage animals were outcrossed to wild-type animals to generate the F4 generation. The outcross (OC) involved an F3 generation male being crossed with a wild-type female and the reverse outcross (ROC) involved an F3 generation female being crossed with a wild-type male. The F4 generation animals were aged to 10 months and then sacrificed to assess transgenerational obesity and associated disease incidence as previously described (Additional file 6: Tables S4 and Additional file 7: Table S5).

The F3 generation LD DDT-induced transgenerational obesity in the female was transmitted with a trend to the F4 generation outcross LD DDT lineage, but was not statistically different (Figure?4a). In contrast, the F3 generation LD DDT-induced transgenerational obesity in the male was transmitted to the F4 generation reverse outcross LD DDT lineage (Figure?4b). Therefore, the obesity in the female appears to be transmitted through the male germline or require both parental germline contributions, while the obesity in the male is transmitted through the female germline. The LD DDT-induced obesity-associated transgenerational testis disease was transmitted to the F4 generation reverse outcross, LD DDT lineage (*P* <0.06) (Figure?4c). Therefore, the testis disease was transmitted through the female germline. The LD DDT-induced obesity-associated transgenerational polycystic ovarian disease was transmitted to the F4 generation reverse outcross LD DDT lineage (Figure?4d). Therefore, the polycystic ovarian disease was also transmitted through the female germline. The LD DDT-induced obesity-associated transgenerational kidney disease in the female was transmitted to the F4 generation reverse outcross LD DDT lineage with a strong trend (*P* <0.09) (Figure?4e). The LD DDT-induced transgenerational kidney disease in the male was transmitted to both the F4 generation outcross and reverse outcross LD DDT lineages (Figure?4f). Therefore, the female kidney disease was transmitted through the female germline, but the male kidney disease was transmitted by both the male and female germlines. Combined observations indicate that the LD DDT-induced transgenerational obesity and associated disease is predominately transmitted through the female (egg) germline, but specific diseases (for example, kidney) are also transmitted through the male (sperm) germline. This is one of the first observations that the female germline can also transmit transgenerational disease. Observations suggest the parental germline origins for the transgenerational disease may be exposure specific and also disease or organ specific.

**Figure 4:**
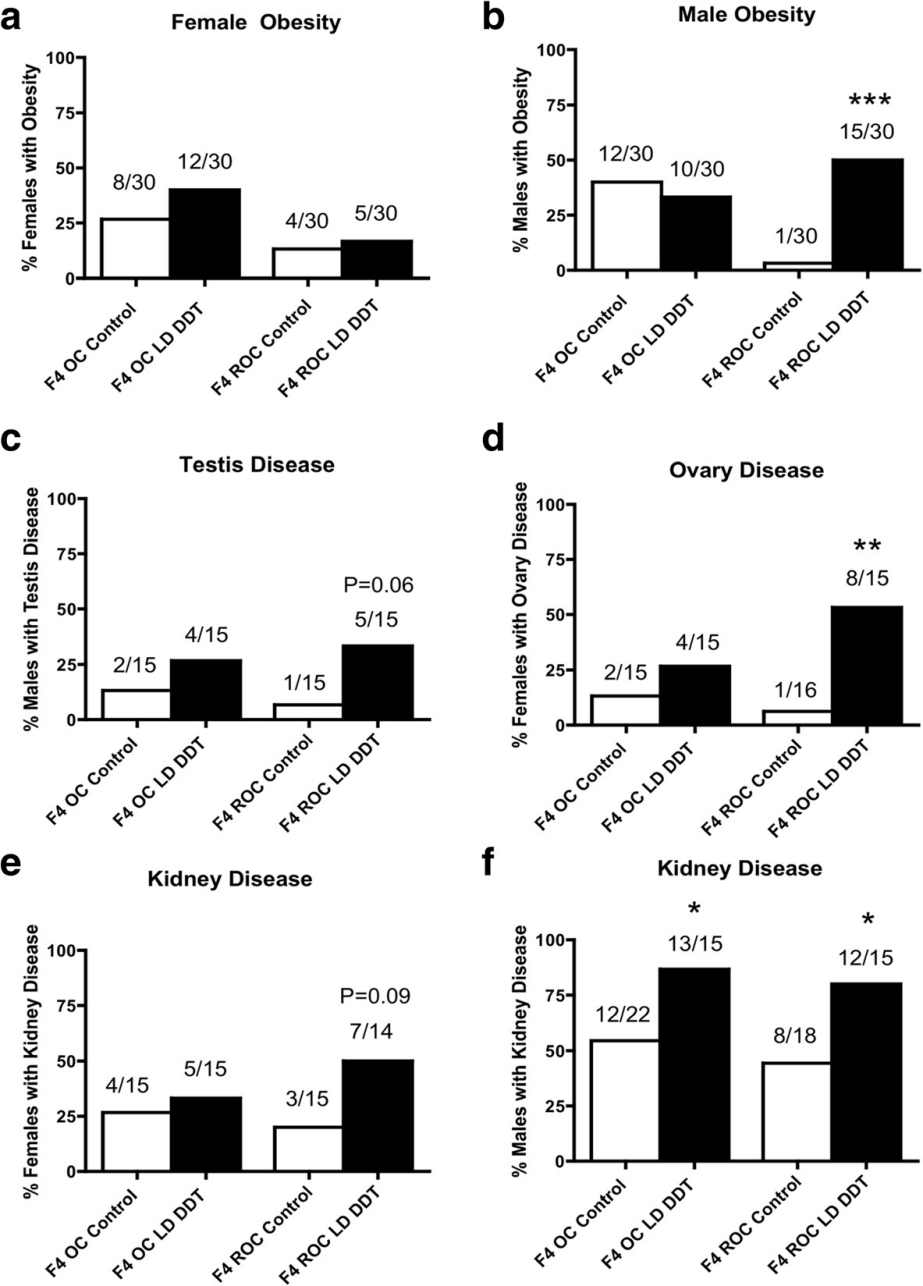
**Transgenerational disease in F4 generation outcross or reverse outcross offspring for both male and female germline transmission.** Incidences of obesity in females **(a)** and in males **(b)**, ovary disease **(c)**, testis disease **(d)**, kidney disease in females **(e)**, in males **(f)**, of the F4 generation outcross (OC) (F3 dichlorodiphenyltrichloroethane (DDT) lineage male cross with wild-type female) or reverse outcross (ROC) (F3 DDT lineage female cross with wild-type male) offspring of the control, DDT, and lower dose (LD) DDT F3 generation lineages. The number of diseased rats/total number of rats (n value) in each lineage are shown above the bar. A logistic regression analysis with *P* value compared to control is indicated (*P <0.05; **P <0.01; ***P <0.001).

### Epigenetic transgenerational transmission of sperm epimutations

The DDT-induced epigenetic transgenerational inheritance of obesity and associated disease requires the germline transmission of epimutations [1-3]. Previously, F3 generation sperm have been shown to have differential DMR induced by vinclozolin [4,57] and a variety of other environmental toxicants [7]. Interestingly, the sperm epimutations induced appear to be unique to the specific environmental exposure [7]. The present study investigated the sperm epimutations induced by DDT and present in the F3 generation sperm. Three different experiments with each involving a different pool of three different animals from different litters were used. The F3 generation control and LD DDT lineage sperm were collected and analyzed using the MeDIP procedure followed by a promoter tiling array chip (MeDIP-chip) analysis as previously described [7,57]. Those DMR between the control and LD DDT lineage sperm samples that were statistically significant (*P* <10^-5^) were identified and termed epimutations. When the DMR were present in all three different experiments they were termed ?intersection? DMR. A total of 39 intersection DMR were identified and their chromosomal locations are indicated in Figure?5a and Additional file 8: Table S6. As previously described [7], the majority (28 DMR) of the intersection DMR were unique to the DDT exposure (Figure?5b) and not common with those epimutations previously identified in vinclozolin, plastics, dioxin, pesticide or hydrocarbon exposures [7,57] (Additional file 8: Table S6). A less stringent analysis uses the mean averages of the three different experiments to identify significantly different DMR termed ?average?. Using a *P* <10^-5^ cut-off a total of 231 average DMR were identified as shown in Figure?5a and Additional file 9: Table S7. Confirmation of the MeDIP-chip analysis DMR data used a quantitative PCR MeDIP-QPCR analysis. Three genes associated with the DMR from the intersection DMR list were selected due to having high interconnectivity in the gene network described below. The genes and statistically significant change (*P* <0.05) between control versus DDT lineage F3 generation sperm MeDIP samples were *Tubb3* (tubulin beta 3) (>13.8 fold change), *Carm1* (coactivator-associated arginine methyltransferase) (>50 fold change), and *Slc4a4* (solute carrier family 4 sodium bicarbonate cotransporter member 4) (>10 fold change) (Additional file 10: Figure S3). A profile of the MeDIP-chip data for these genes is presented and compared to the MeDIP-QPCR data (Additional file 10: Figure S3). Therefore, the MeDIP-QPCR confirmed the MeDIP-chip analysis for these DDT-induced sperm DMR. Observations demonstrate DDT induced a unique set of epimutations in the F3 generation sperm.

**Figure 5:**
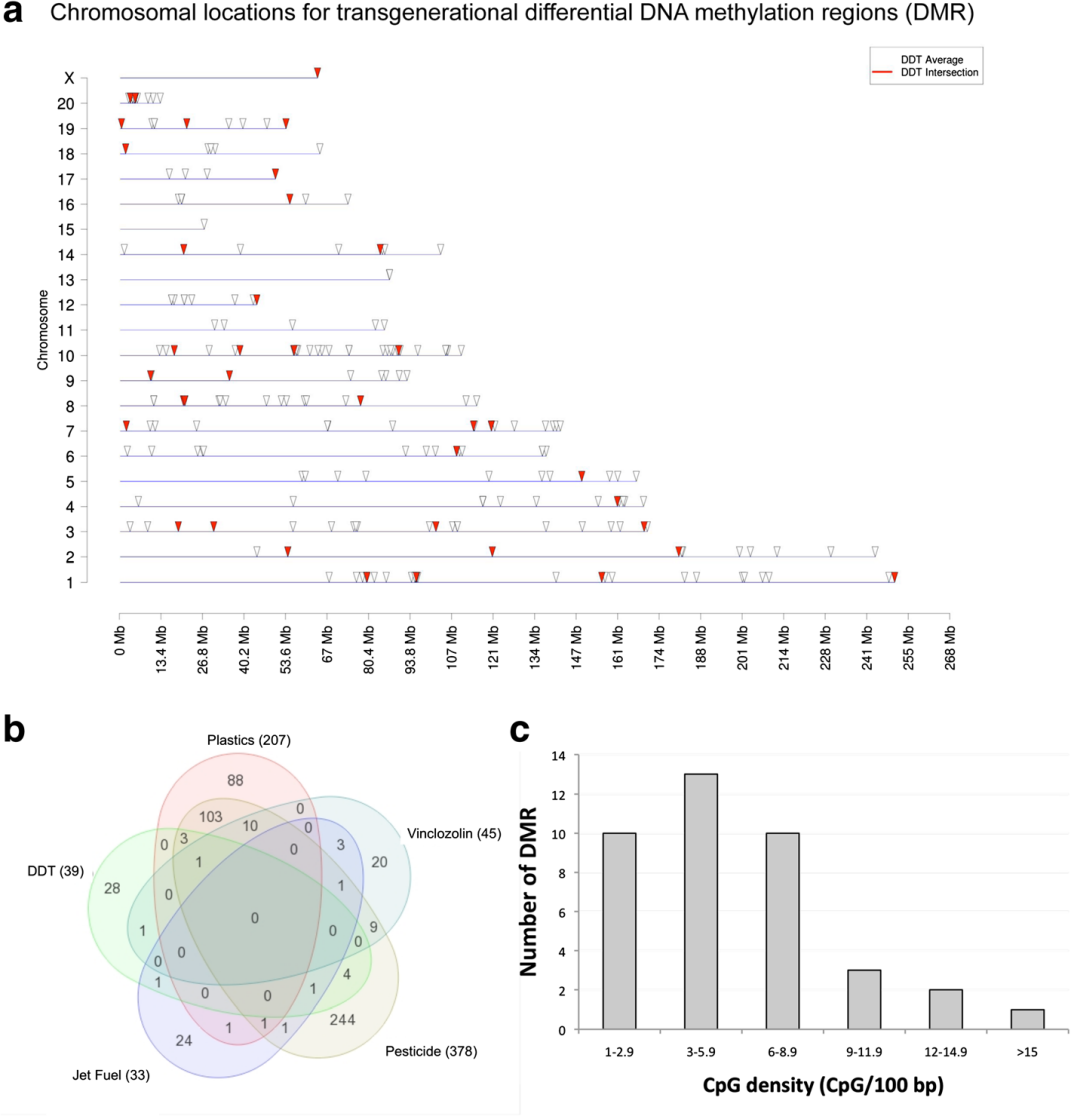
**Transgenerational F3 generation sperm epimutations. (a)** Chromosomal locations for differential DNA methylated regions (DMR) in sperm DNA from F3 generation dichlorodiphenyltrichloroethane (DDT) lineage rats compared to control lineage (arrowheads). The high stringency intersection epimutations are identified as red arrows, which is a subset of the less stringent ?average? DMR indicated with open arrows. The chromosomal size and number are listed (Additional file 8: Table S6 and Additional file: 9 Table S7). **(b)** A Venn diagram of DMR from various F3 generation exposure lineages including: vinclozolin, plastics, pesticides, hydrocarbons and DDT. The total number of DMR per exposure lineage in brackets is presented and unique and overlapping DMR identified. **(c)** The CpG/100 bp is presented and the corresponding number of DMR associated. The density is presented as the number of CpG for 100 bp of the DMR.

A previous study identified a unique genomic feature associated with the environmentally induced sperm DMR [7,57]. The genomic feature identified in all transgenerational sperm DMR previously identified is a low-density CpG of less than 10 CpG/100 bp [7,57]. These low-density regions are termed ?CpG deserts? that contain clusters of CpG [7]. The DDT transgenerational sperm DMR were found to contain less than 15 CpG/100 bp, with 3 to 6 CpG/100 bp being the most predominant density (Figure?5c). Therefore, the low-density ?CpG desert? genomic feature was a component of the DDT F3 generation sperm DMR.

Further bioinformatic analysis of the genes associated with the DMR in Table S7 identified a network of interconnected genes using a literature-based analysis (see Methods). A number of highly connected genes were identified that are involved in extracellular, signaling and transcriptional activities (Figure?6a). The potential role of this gene network or specific signaling processes in the DDT promoted epigenetic transgenerational inheritance of disease remains to be elucidated. The final analysis of the DMR-associated genes was to correlate specific genes known to be associated with obesity with the sperm DMR. The known obesity-associated genes had 13 correlated DMR (Figure?6b). The known obesity-associated genes also had three genes having correlation with genes known to be associated with polycystic ovarian disease. Therefore, a number of previously identified obesity-associated genes correlated with the epimutations identified.

**Figure 6:**
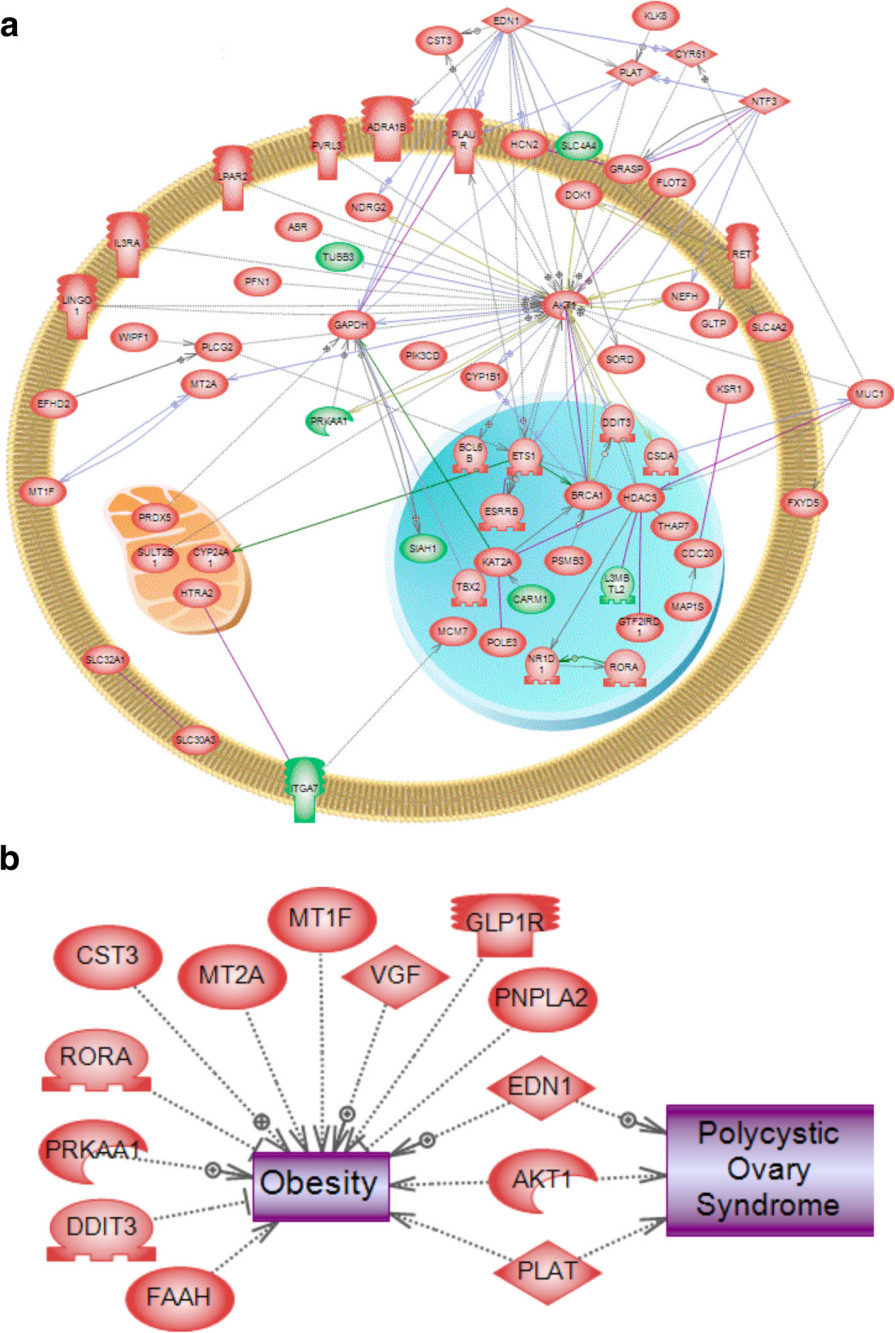
**Transgenerational F3 generation sperm epimutation-associated gene network. (a)** The gene network associated with the differential DNA methylation regions (epimutations) with cellular location indicated. The green labeled genes are those in the high stringency intersection set of 38 differential DNA methylated region (DMR) associated genes. **(b)** The genes that have a known link to obesity and polycystic ovarian disease that are associated with dichlorodiphenyltrichloroethane (DDT) induced sperm DMRgenes are presented.

## Discussion

The present study demonstrates that ancestral DDT exposure during a critical window of germline development can promote the epigenetic transgenerational inheritance of obesity and a number of associated complex disease traits. The doses of DDT used have been shown to be wildlife and human environmental exposure levels [22,58]. The human DDT lowest observed adverse effect level LOAEL is 0.25 mg/kg/day which is lower than that used in the present study. The objective of the present study was to determine the potential transgenerational actions of DDT, such that a higher dose was used initially. We administered through an intraperitoneal injection to determine the potential to promote phenotypes. Previous studies have shown intraperitoneal injection is less stressful than oral gavage and less problematic to promote indirect effects from mode of administration [65]. Therefore, the present study was not designed as a risk assessment analysis for DDT exposures, but to determine the potential ability of DDT to promote transgenerational phenotypes. Future research can now use the information obtained to design risk assessment studies with more appropriate modes of administration for environmental doses. However, the observations do demonstrate the ability of ancestral DDT exposures to promote transgenerational adult onset obesity and associated disease.

The most dramatic increase in the incidence of adult onset disease observed was with obesity and was only in the F3 generation. Over 50% of the males and females with ancestral DDT exposure developed an obesity condition of increased body weight and abdominal adiposity. Previous reviews of the definition and cause for obesity suggest that weight gain, adiposity and presence of other obesity-associated disease is sufficient to refer to the pathology observed as obesity [28,36,49-51]. The US Centers for Disease Control in 2010 reported that 33% of adults in the US are obese and 17% of children between ages 2 to 19 are obese. Obesity is a contributing factor and/or precursor for many other diseases including polycystic ovarian disease, testis disease, kidney disease, liver disease, cardiovascular disease, type 2 diabetes, and diminished average life expectancy [66] (Figure?2). The dramatic increase in obesity over the past 50 years suggests that environmental factors are important in the disease etiology. The primary causal factor suggested is overnutrition [28,67,68]. However, recent studies have suggested environmental toxicants such as plastics [31], jet fuel hydrocarbons [32], and tributyltin [33] can promote transgenerational obesity in rodents. Waterland and colleagues suggested that epigenetic mechanisms are involved in transgenerational transmission of maternal obesity [10]. The present study demonstrated the epigenetic transgenerational inheritance of obesity in both males and females following an ancestral DDT exposure. The two obesity conditions of weight gain and abdominal adiposity are sufficient to identify obesity [28,49,50], however, future studies will need to assess the effects on other obesity parameters such as bone mineralization, body length and metabolic disease. Interestingly, several obesity-associated diseases were identified in the present study including polycystic ovarian disease, testis disease and kidney disease [9,27,44,45,69-72]. The obesity observed in humans and rodents have similar disease phenotypes and associations [28,36]. The functional correlation of these diseases needs to be elucidated. Interestingly, the DDT-induced obesity was only observed in the F3 generation and not the F1 direct exposure generation. The molecular mechanisms involved in the F1 generation and F3 generation are distinct [1]. The F1 generation pathology is due to direct exposure of the somatic cells of the fetus and does not involve the germline. The F3 generation pathology is due to a transgenerational germline mediated mechanism [1]. The epigenetic transgenerational inheritance of obesity in the F3 generation is distinct from the somatic exposure mechanism of the F1 generation [1,2]. Therefore, some germline mediated detrimental effects of DDT could be hidden for several generations before they become apparent. Observations suggest ancestral exposures to DDT may be a component of the rising incidence of obesity observed in the current human population. Since the primary human exposures to DDT in the US occurred in the 1950s, three generations have developed with a progressively increasing incidence of obesity in the population. Therefore, future studies are required to assess the importance of ancestral DDT exposures to the etiology of obesity.

The transgenerational obesity-associated diseases and abnormalities (Figure?2) observed include testis disease, polycystic ovarian disease, immune abnormalities and kidney disease. Direct DDT exposure has been shown to promote testis disease in rats (7.5 mg/kg/day for 36 weeks) [22], alligators [58], and fish [73]. Interestingly, male offspring of women exposed to DDT during pregnancy had an increase in testis disease observed 30 years following the exposure [74]. Polycystic ovarian disease is now the most common reproductive disease in women leading to infertility and endocrine abnormalities [75]. A number of environmental compounds have the ability to promote transgenerational ovarian disease in rats [60]. Kidney disease has been shown to be induced following direct DDT exposure in rats (10 mg/kg/day for 27 days) [22] and in humans [76]. A number of environmental toxicants can promote transgenerational kidney disease in rats [7]. All these diseases (that is, testis, ovary, and kidney) have increased dramatically over the past decades suggesting a potential environmental component. The presence of these associated diseases in the present study supports the conclusion that DDT exposure of a gestating female promotes the transgenerational inheritance of obesity and associated complex disease traits.

The environmentally induced epigenetic transgenerational inheritance of disease requires the germline transmission of epimutations to subsequent generations [1-4]. The present study identified the DDT-induced transgenerational epimutations in the sperm of the F3 generation males. A comparison of these differential DMR with DMR induced by other environmental toxicants [7] demonstrated a unique DDT DMR signature that may be used to assess ancestral DDT exposure. The DDT transgenerational DMR had a low-density CpG ?CpG desert? genomic feature, as previously described with other exposures [7,57]. These low-density CpG deserts containing clusters of CpG are speculated to be a regulatory genomic feature associated with the DDT DMR. Further studies are needed to elucidate the functional significance of this DMR genomic feature. The presence of the F3 generation DMR demonstrates a transgenerational transmission of an epigenetic alteration in the germline. Future studies will need to compare the F3 generation DMR with the F1 and F2 generations. The present study demonstrates the epigenetic transmission and inheritance of sperm epimutations.

Analysis of the genes associated with the DDT sperm DMR identified a number of genes previously shown to be involved in obesity or the associated polycystic ovarian disease (Figure?6). The role of the DMR identified in the regulation of these correlated genes remains to be elucidated, however, these genes have previously been shown to be associated with obesity. Observations provide potential mechanistic links with the pathologies observed. Previous studies have shown that over 50% of females with polycystic ovarian disease are over weight or obese [45,69,70]. Therefore, a strong link exists between obesity and ovarian disease [69]. Interestingly, the transmission of many of the DDT-induced disease states was found to be through the female germline. This is the first female-germline-transmitted transgenerational phenotype identified. The imprinted-like nature of the transgenerational phenotype [2] suggests a parent-of-origin allele specific transmission of disease, which has been shown to be either paternal or maternal. This molecular mechanism needs to be elucidated in future studies, but the present study demonstrates sex specific germline transmission of transgenerational phenotypes. Future studies are now needed to assess the DMR in the egg induced by DDT. Observations suggest a potential exposure and disease specificity to the parental origins of the transgenerational phenomena.

## Conclusions

Over 50 years have passed since the publication of the book *Silent Spring* by Rachel Carson that argued the hazards of DDT to wildlife and human health [77]. Since the Gates Foundation and World Health Organization (WHO) have now promoted the use of DDT in Africa and other developing countries for malaria control, the potential hazards of current day exposures now need to be considered in light of the transgenerational actions of DDT. Although the number of lives saved from malaria is significant, the long-term health and economic effects on survivors [78] and subsequent generations also need to be considered. Since other options exist with less toxic shorter half-life pesticides, a more careful risk/benefit consideration of the use of DDT is now needed.

The degree that environmentally induced epigenetic transgenerational inheritance is involved in human obesity and disease etiology is not known. However, since the majority of chronic diseases have increased dramatically over the past decades, environmental exposures and transgenerational epigenetics will likely be a component of disease etiology to seriously consider in the future. A more thorough and mechanistic understanding of the molecular etiology of disease, including the role of environmental epigenetics, is anticipated to provide insights into new diagnostics and therapeutics for specific diseases.

## Abbreviations

BPA: Bisphenol-A; DDE: Dichlorodiphenyldichloroethylene; DDT: Dichlorodiphenyltrichloroethane; DMR: Differential DNA methylation regions; LD: Lower dose; MeDIP: Methylated DNA immunoprecipitation.

## Competing interests

The authors declare they have no competing interests.

## Authors? contributions

MKS conceived and designed the study. MM, RT, MMH, EEN and CG-B performed the experiments and acquired the data. All authors analyzed the data. MKS and MM wrote the manuscript. All authors edited the manuscript. All authors read and approved the final manuscript.

## Supplementary Material

Additional file 1: Table S1 **(A)** Body weight and organ weights in F1 and F3 generation female rats of control, dichlorodiphenyltrichloroethane (DDT) and lower dose DDT lineages (mean???standard error). **(B)** Body weight (g) and organ weights (% of body weight) in F1 and F3 generation male rats of control, DDT and lower dose DDT (mean???standard error). Asterisks (*, **, ***), if present, indicate statistically significant differences using a *t* test between 3 means of control and DDT or low dose DDT lineages (*P* <0.05, *P* <0.01 and *P* <0.001, respectively); ND?=?not determined. Click here for file

Additional file 2: Figure S1 Histopathology of transgenerational disease. **(A)** The testis and prostate histopathology. **(B)** The male and female kidney histopathology. The F3 generation control lineage **(A)**, dichlorodiphenyltrichloroethane (DDT) lineage **(B)**, and lower dose DDT lineage **(C)** for each tissue presented. The bar is 100 ?m and insets of higher magnification show the various pathologies described. Click here for file

Additional file 4: Table S2 **(A)** Individual disease incidence in F1 generation female rats of control, dichlorodiphenyltrichloroethane (DDT) and lower dose DDT lineages. **(B)** Individual disease incidence in F1 generation male rats of control, DDT and lower dose DDT lineages. ?+? indicates the presence and ?-? indicates the absence of disease; a blank cell indicates ?not determined?. Animal IDs with a ?C? belong to the control group, those with a ?D? belong to the DDT group and those with ?LD? belong to the lower dose DDT group. See Methods section for disease assessment in rats. The number of animals per litter (litter representation) mean???SEM used for each specific disease/abnormality assessment within the control, DDT or lower dose DDT lineages were not found to be statistically different (*P* >0.05), so no litter bias was detected. Click here for file

Additional file 5: Table S3 **(A)** Individual disease incidence in F3 generation female rats of control, dichlorodiphenyltrichloroethane (DDT) and lower dose DDT lineages. **(B)** Individual disease incidence in F3 generation male rats of control, DDT and lower dose DDT lineages. ?+? indicates the presence and ?-? indicates the absence of disease; a blank cell indicates ?not determined?. Animal IDs with a ?C? belong to the control group, those with a ?D? belong to the DDT group and those with ?LD? belong to the lower dose DDT group. See Methods section for disease assessment in rats. The number of animals per litter (litter representation) mean???SEM used for each specific disease/abnormality assessment within the control, DDT or lower dose DDT lineages were not found to be statistically different (*P* >0.05), so no litter bias was detected. Click here for file

Additional file 3: Figure S2 Transgenerational physiological and disease incidence in the F1 and F3 generation. Prostate disease **(A)** and uterine infection **(B)** are presented. Serum estradiol concentrations in proestrus-estrus in F3 generation control, dichlorodiphenyltrichloroethane (DDT) and lower dose DDT lineage females **(C)**. Serum estradiol concentrations in diestrus in F3 generation control, DDT and low dose DDT lineage females **(D)**. Serum testosterone concentrations in the F3 generation control, DDT and low dose DDT lineage males **(E)**. Pubertal abnormalities in female **(F)** and male **(G)** animals. Tumor development in female **(H)** and male **(I)** animals from the F1 and F3 generation control, DDT, and low DDT dose lineages. The number of disease rates/total numbers of rats (n value) in each lineage are shown above the bars. Those showing numbers above the bars were analyzed with logistic regression analysis and those with a mean???SEM indicated were analyzed with a *t* test with the *P* value represented (*P <0.05; **P <0.01; ***P <0.001). Click here for file

Additional file 6: Table S4 **(A)** Individual disease incidence in F4 generation outcross female rats of control and lower dose dichlorodiphenyltrichloroethane (DDT) lineages. **(B)** Individual disease incidence in F4 generation outcross male rats of control and lower dose DDT lineages. ?+? indicates the presence and ?-? indicates the absence of disease; a blank cell indicates ?not determined?. Animal IDs with a ?C? belong to the control group, those with a ?LD? belong to the lower dose DDT group. See Methods section for disease assessment in rats. The number of animals per litter (litter representation) mean???SEM used for each specific disease/abnormality assessment within the control or lower dose DDT lineages were not found to be statistically different (*P* >0.05), so no litter bias was detected. Click here for file

Additional file 7: Table S5 **(A)** Individual disease incidence in F4 generation reverse outcross female rats of control and lower dose dichlorodiphenyltrichloroethane (DDT) lineages. **(B)** Individual disease incidence in F4 generation Reverse Outcross male rats of Control and Lower Dose DDT lineages. ?+? indicates the presence and ?-? indicates the absence of disease; a blank cell indicates ?not determined?. Animal IDs with a ?C? belong to the control group, those with a ?LD? belong to the lower dose DDT group. See Methods section for disease assessment in rats. The number of animals per litter (litter representation) mean???SEM used for each specific disease/abnormality assessment within the control or lower dose DDT lineages were not found to be statistically different (*P* >0.05), so no litter bias was detected. Click here for file

Additional file 8: Table S6 Dichlorodiphenyltrichloroethane (DDT) induced F3 generation sperm differential DNA methylation regions (DMR) (intersection). Epimutations found in F3-generation sperm after exposure of F0 generation gestating females to DDT, obtained by intersection of the five results of three methylated DNA fragment immunoprecipitation (MeDIP)-chip comparative hybridizations. The genes in bold were found to be unique DMR associated only with DDT-induced DMR. Click here for file

Additional file 9: Table S7 Dichlorodiphenyltrichloroethane (DDT) induced F3 generation sperm differential DNA methylation regions (DMR) (average). Average found in F3-generation rat sperm after exposure of F0 generation to DDT, obtained averaging the results of three comparative hybridizations. Click here for file

Additional file 10: Figure S3 Immunoprecipitation of methylated DNA fragments-quantitative polymerase chain reaction (MeDIP-QPCR) confirmation of selected differentially methylated DNA regions (DMR). Confirmation of MeDIP-chip identified DMR with an MeDIP-QPCR analysis. **(A)** The DMR-associated genes *Tubb3*, *Carm1*, and *Slc4c4* were selected and QPCR with a real-time PCR analysis on MeDIP samples from control and DDT lineage sperm samples performed. The relative changes (DDT/control) are presented with the asterisks (*) indicating statistical differences *P* <0.05. The MeDIP-chip profiles for **(B)***Carm1* DMR, **(C)***Slc4c4* DMR, and **(D)***Tubb3* DMR are presented with the bars indicating individual oligonucleotides probes and chromosomal location. The top gray bar represents the DDT lineage F3 generation sperm MeDIP sample hybridization and the bottom black bar represents the control lineage F3 generation sperm MeDIP sample hybridization. The region with an asterisk (*) above the bar represents statistical (*P* <0.05) alterations with an increase in DDT MeDIP sample hybridization versus control. The data represent the mean of three different experiments and associated samples and the MeDIP-chip profiles are a representative hybridization profile. Click here for file
