# Supplementary material for: Ancestral dichlorodiphenyltrichloroethane (DDT) exposure promotes epigenetic transgenerational inheritance of obesity
Source: BMC Med. 2013 Oct 23;11:228. doi: 10.1186/1741-7015-11-228 (PMC3853586; doi:10.1186/1741-7015-11-228)
Supplement: Additional file 1: Table S1 — (A) Body weight and organ weights in F1 and F3 generation female rats of control, dichlorodiphenyltrichloroethane (DDT) and lower dose DDT lineages (mean???standard error). (B) Body weight (g) and organ weights (% of body weight) in F1 and F3 generation male rats of control, DDT and lower dose DDT (mean???standard error). Asterisks (*, **, ***), if present, indicate statistically significant differences using a t test between 3 means of control and DDT or low dose DDT lineages (P <0.05, P <0.01 and P <0.001, respectively); ND?=?not determined. [file 1741-7015-11-228-S1.pdf]

Supplemental Table S1A.

Female rat body weight and organ weights in F1 and F3 generation female rats Control, DDT and Low Dose DDT lineages (mean ± standard error).

Generation	Treatment	Sex	Body Weight (grams)	Ovaries (% BW)	Uterus (% BW)	Kidney (% BW)
F1	Control	F	323.0 ± 6.67	0.0492 ± 0.0012	0.2832 ± 0.0191	0.3289 ± 0.0054
F1	DDT	F	295.6** ± 6.76	0.0541 ± 0.0027	0.3349 ± 0.0245	0.3499* ± 0.0083
F1	Low Dose DDT	F	298.9** ± 4.58	0.0528 ± 0.0023	0.3569* ± 0.0299	0.3649*** ± 0.0053
F3	Control	F	289.3 ± 5.01	nd	nd	nd
F3	DDT	F	269.5** ± 3.52	nd	nd	nd
F3	Low Dose DDT	F	287.4 ± 3.01	nd	nd	nd

Supplemental Table S1B.

Male rat body weight (grams) and organ weights (% of body weight) in F1 and F3 generation Control, DDT and Low Dose DDT (mean ± standard error).

Generation	Treatment	Sex	Body Weight (grams)	Testis (% BW)	Prostate (% BW)	Seminal Vesicle (% BW)	Epididymis (% BW)	Kidney (% BW)
F1	Control	M	567.2 ± 6.83	0.7799 ± 0.0127	0.1943 ± 0.0078	0.0835 ± 0.0020	0.2566 ± 0.0037	0.3415 ± 0.0077
F1	DDT	M	518.3** ± 12.84	0.7661 ± 0.0193	0.1894 ± 0.0083	0.0873 ± 0.0056	0.2529 ± 0.0072	0.3725* ± 0.0120
F1	Low Dose DDT	M	546.6 ± 10.22	0.7781 ± 0.0127	0.1965 ± 0.0070	0.0851 ± 0.0025	0.2716* ± 0.0047	0.3834** ± 0.0107
F3	Control	M	513.7 ± 8.79	nd	nd	nd	nd	nd
F3	DDT	M	496.0 ± 7.39	nd	nd	nd	nd	nd
F3	Low dose DDT	M	522.2 ± 9.34	nd	nd	nd	nd	nd

Asterisks (*, **, ***), if present, indicate statistically significant differences between means of control and DDT or low dose DDT lineages (P<0.05, P<0.01 and P<0.001 respectively); the nd indicates not determined and tissue weights were only examined in the F1 generation to assess potential toxicity.
