# Supplementary figures and images for: Ancestral dichlorodiphenyltrichloroethane (DDT) exposure promotes epigenetic transgenerational inheritance of obesity
Source: BMC Med. 2013 Oct 23;11:228. doi: 10.1186/1741-7015-11-228 (PMC3853586; doi:10.1186/1741-7015-11-228)

Supplemental Figure S1A (Color)

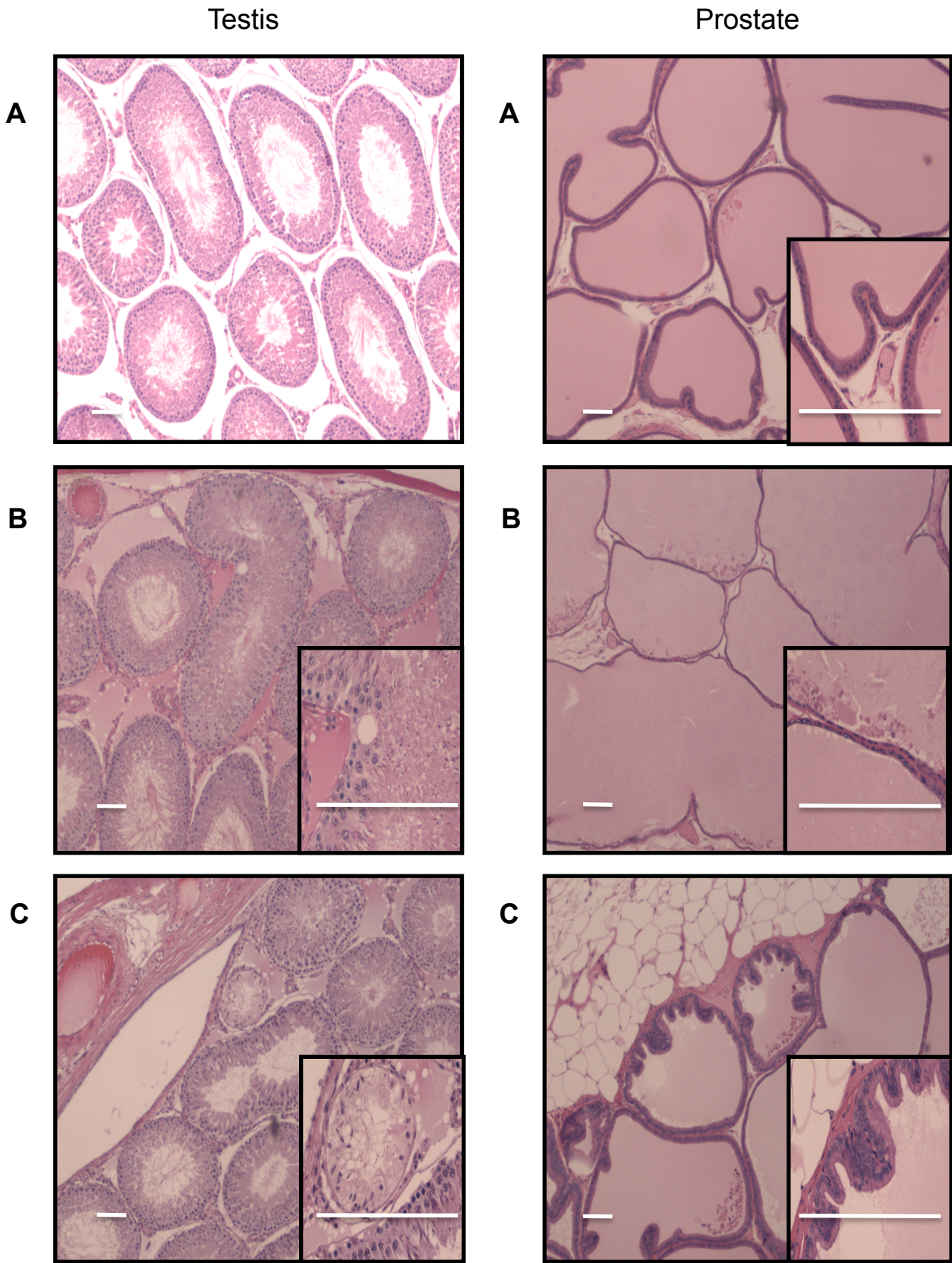

Supplement: Additional file 2: Figure S1 — Histopathology of transgenerational disease. (A) The testis and prostate histopathology. (B) The male and female kidney histopathology. The F3 generation control lineage (A), dichlorodiphenyltrichloroethane (DDT) lineage (B), and lower dose DDT lineage (C) for each tissue presented. The bar is 100 ?m and insets of higher magnification show the various pathologies described. [file 1741-7015-11-228-S2.zip › 3183211929952274_add2a.pdf]

Supplemental Figure S1B (Color)

Female Kidney

Male Kidney

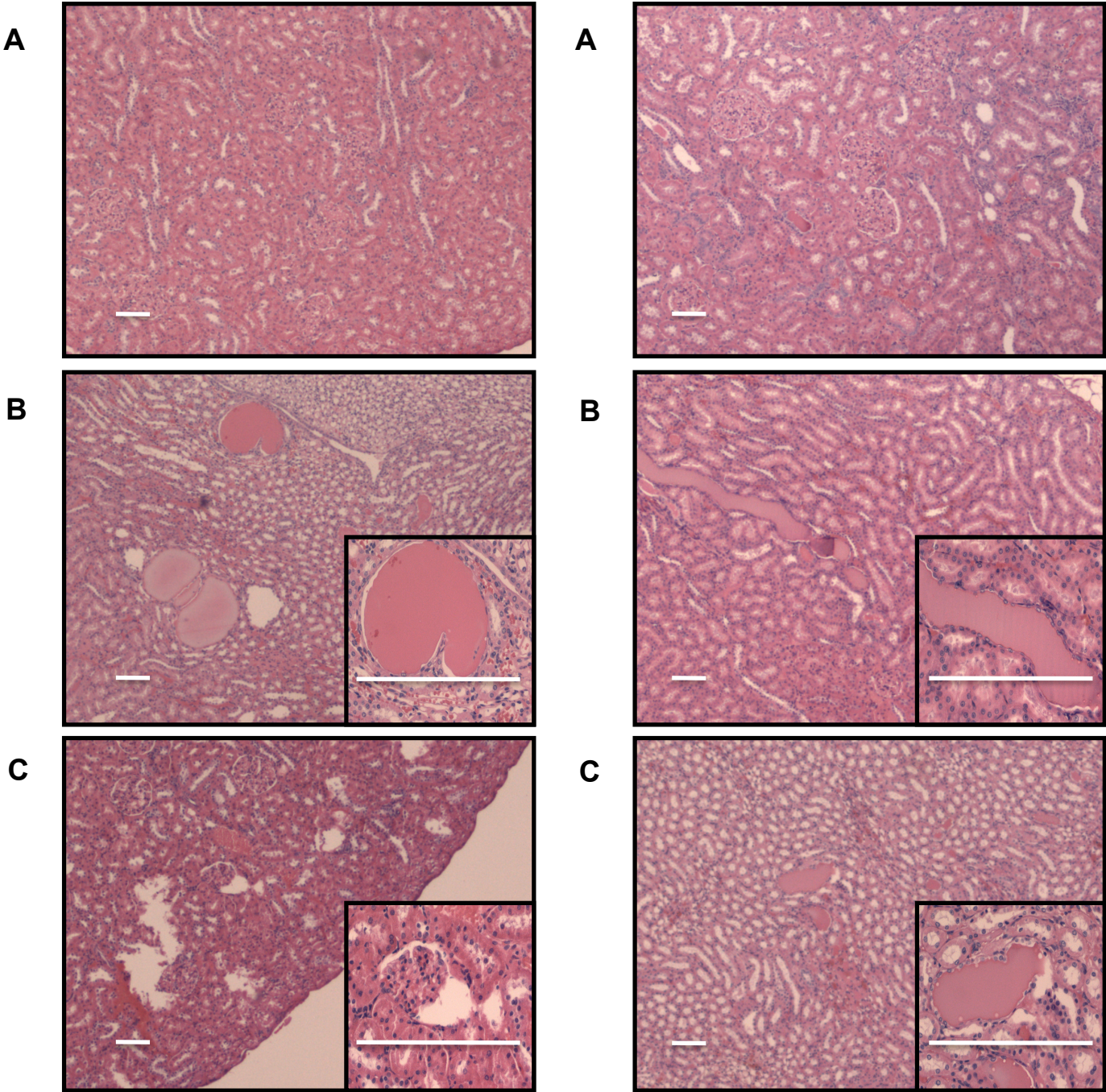

Supplement: Additional file 2: Figure S1 — Histopathology of transgenerational disease. (A) The testis and prostate histopathology. (B) The male and female kidney histopathology. The F3 generation control lineage (A), dichlorodiphenyltrichloroethane (DDT) lineage (B), and lower dose DDT lineage (C) for each tissue presented. The bar is 100 ?m and insets of higher magnification show the various pathologies described. [file 1741-7015-11-228-S2.zip › 3183211929952274_add2b.pdf]

Supplemental Figure S2

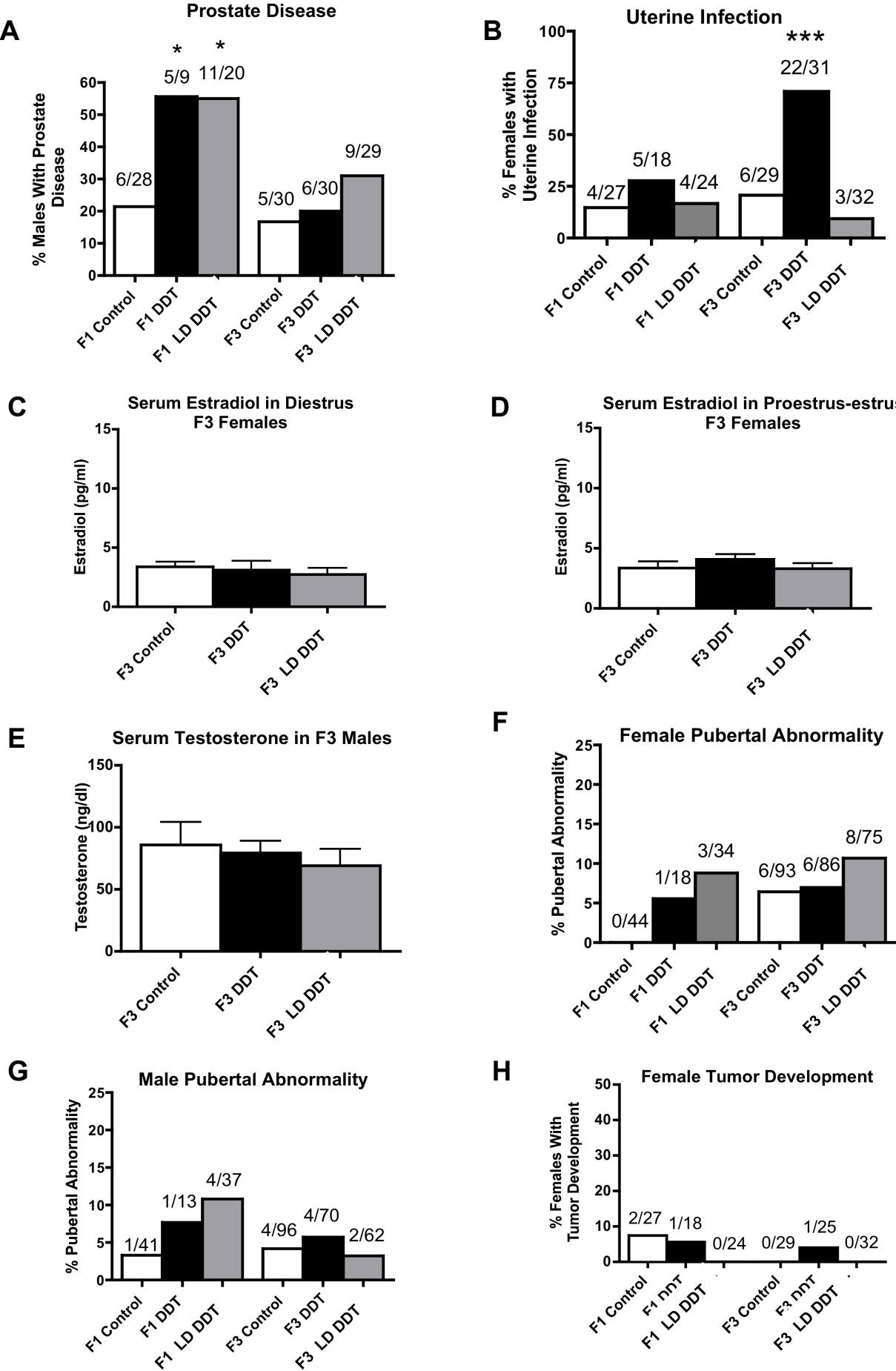

Supplemental Figure S2 (continued)

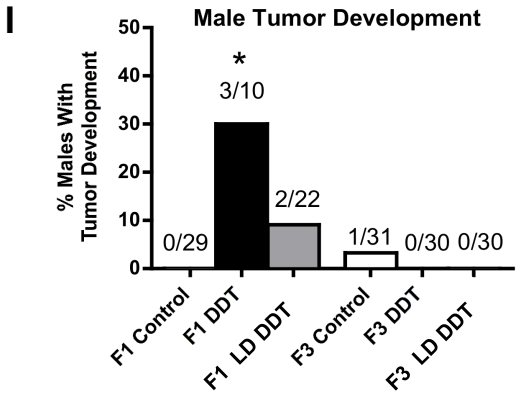

Supplement: Additional file 3: Figure S2 — Transgenerational physiological and disease incidence in the F1 and F3 generation. Prostate disease (A) and uterine infection (B) are presented. Serum estradiol concentrations in proestrus-estrus in F3 generation control, dichlorodiphenyltrichloroethane (DDT) and lower dose DDT lineage females (C). Serum estradiol concentrations in diestrus in F3 generation control, DDT and low dose DDT lineage females (D). Serum testosterone concentrations in the F3 generation control, DDT and low dose DDT lineage males (E). Pubertal abnormalities in female (F) and male (G) animals. Tumor development in female (H) and male (I) animals from the F1 and F3 generation control, DDT, and low DDT dose lineages. The number of disease rates/total numbers of rats (n value) in each lineage are shown above the bars. Those showing numbers above the bars were analyzed with logistic regression analysis and those with a mean???SEM indicated were analyzed with a t test with the P value represented (*P <0.05; **P <0.01; ***P <0.001). [file 1741-7015-11-228-S3.pdf]

Supplemental Figure S3

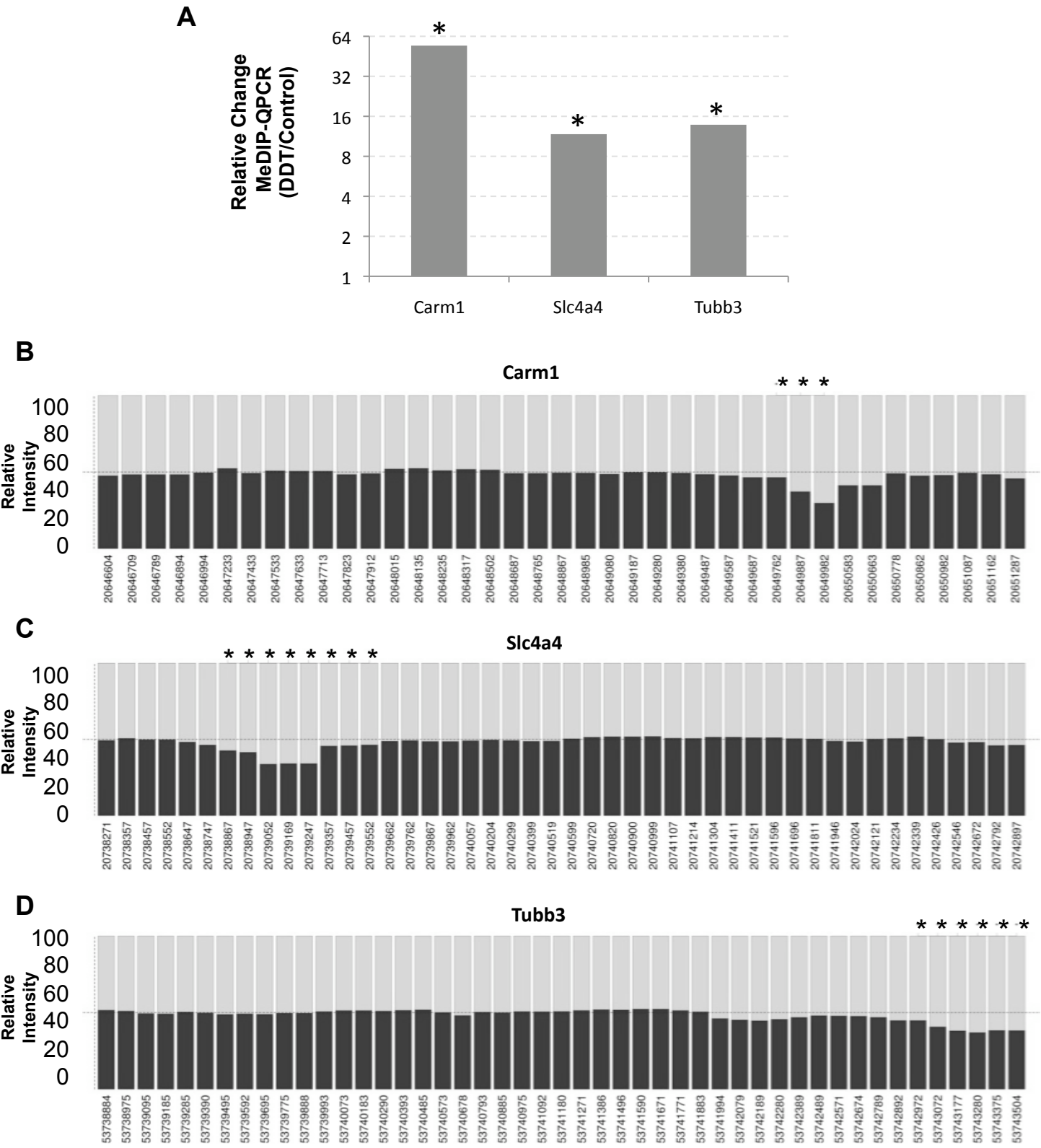

Supplement: Additional file 10: Figure S3 — Immunoprecipitation of methylated DNA fragments-quantitative polymerase chain reaction (MeDIP-QPCR) confirmation of selected differentially methylated DNA regions (DMR). Confirmation of MeDIP-chip identified DMR with an MeDIP-QPCR analysis. (A) The DMR-associated genes Tubb3, Carm1, and Slc4c4 were selected and QPCR with a real-time PCR analysis on MeDIP samples from control and DDT lineage sperm samples performed. The relative changes (DDT/control) are presented with the asterisks (*) indicating statistical differences P <0.05. The MeDIP-chip profiles for (B)Carm1 DMR, (C)Slc4c4 DMR, and (D)Tubb3 DMR are presented with the bars indicating individual oligonucleotides probes and chromosomal location. The top gray bar represents the DDT lineage F3 generation sperm MeDIP sample hybridization and the bottom black bar represents the control lineage F3 generation sperm MeDIP sample hybridization. The region with an asterisk (*) above the bar represents statistical (P <0.05) alterations with an increase in DDT MeDIP sample hybridization versus control. The data represent the mean of three different experiments and associated samples and the MeDIP-chip profiles are a representative hybridization profile. [file 1741-7015-11-228-S10.pdf]
