# Supplementary material for: Ancestral dichlorodiphenyltrichloroethane (DDT) exposure promotes epigenetic transgenerational inheritance of obesity
Source: BMC Med. 2013 Oct 23;11:228. doi: 10.1186/1741-7015-11-228 (PMC3853586; doi:10.1186/1741-7015-11-228)
Supplement: Additional file 4: Table S2 — (A) Individual disease incidence in F1 generation female rats of control, dichlorodiphenyltrichloroethane (DDT) and lower dose DDT lineages. (B) Individual disease incidence in F1 generation male rats of control, DDT and lower dose DDT lineages. ?+? indicates the presence and ?-? indicates the absence of disease; a blank cell indicates ?not determined?. Animal IDs with a ?C? belong to the control group, those with a ?D? belong to the DDT group and those with ?LD? belong to the lower dose DDT group. See Methods section for disease assessment in rats. The number of animals per litter (litter representation) mean???SEM used for each specific disease/abnormality assessment within the control, DDT or lower dose DDT lineages were not found to be statistically different (P >0.05), so no litter bias was detected. [file 1741-7015-11-228-S4.pdf]

Supplemental Table S2A.

Individual disease incidence in F1 generation female rats of Control, DDT and Lower Dose DDT lineages.

Serial Number	Rat ID	Puberty	Ovary	Uterus	Kidney	Obesity	Tumor	Total Disease
C1	MCTT2-1-1-1	-		-	-	-	-	
C2	MCTT2-1-1-2	-		+	-	-	-	1
C3	MCWW0-1-2-1	-		-		-	+	1
C4	MCWW0-1-2-2	-	-	-	-	-	-	
C5	MCWW0-1-2-3	-	-	-	-	-	-	
C6	MCWW0-1-2-4	-	-	-	-	-	-	
C7	MCAA0-1-3-1	-	-	-	-	-	-	
C8	MCAA0-1-3-2	-		-	-	-	-	
C9	MCAA0-1-3-3	-	-	+	-	-	-	1
C10	MCAA0-1-3-4	-		+	-	-	-	1
C11	MCGG1-1-4-1	-	-	-	-	+	-	1
C12	MCGG1-1-4-2	-	-	-		+	-	1
C13	MCGG1-1-4-3	-	-	-		-	+	1
C14	MCGG1-1-4-4	-	-	-		-	-	
C15	MCWW0-1-6-1	-	-	-	-	-	-	
C16	MCWW0-1-6-2	-		-	-	-	-	
C17	MCWW0-1-6-3	-		-	-	-	-	
C18	MCWW0-1-6-4	-		-	-	-	-	
C19	MCGG2-1-7-1	-		-	-	-	-	
C20	MCGG2-1-7-2	-		-	-	-	-	
C21	MCGG2-1-7-3	-		-	-	-	-	
C22	MCGG2-1-7-4	-		-	+	-	-	1
C23	MCZZ0-1-8-1	-		-	-	-	-	
C24	MCZZ0-1-8-2	-		+	+	-	-	2
C25	MCZZ0-1-8-3	-		-	-	+	-	1
C26	MCZZ0-1-8-4	-		-	-	-	-	
C27	MCZZ0-1-8-5	-		-	-	+	-	1

Serial Number	Rat ID	Puberty	Ovary	Uterus	Kidney	Obesity	Tumor	Total Disease
D1	MDLL2-1-1-1	-	-	-	+	-	-	1
D2	MDLL2-1-1-2	-	+	+	+	-	-	3
D3	MDLL2-1-1-3	-	+	-	-	-	-	1
D4	MDLL2-1-1-4	+		-		+	+	3
D5	MDAA1-1-2-1	-		-	-	-	-	
D6	MDAA1-1-2-2	-		-	-	-	-	
D7	MDAA1-1-2-3	-		-	-	-	-	
D8	MDAA1-1-2-4	-		-	-	-	-	
D9	MDAA1-1-2-5	-		-	-	-	-	
D10	MDGG0-1-3-1	-	+	-	-	-	-	1
D11	MDGG0-1-3-2	-	-	+	+	-	-	2
D12	MDGG0-1-3-3	-	-	+	-	-	-	1
D13	MDGG0-1-3-4	-	+	-	-	-	-	1

D14	MDGG0-1-3-5	-	-	-	+	-	-	1
D15	MDGG0-1-3-6	-	+	+	-	-	-	2
D16	MDGG0-1-3-7	-	-	+	+	-	-	2
D17	MDGG0-1-3-8	-		-	+	-	-	1
D18	MDGG0-1-3-9	-		-	+	-	-	1

Serial Number	Rat ID	Puberty	Ovary	Uterus	Kidney	Obesity	Tumor	Total Disease
LD1	MLDLL0-1-1-2	-		-	-	-	-	
LD2	MLDKK0-1-2-3	-	+	-		-	-	1
LD3	MLDJJ2-1-3-1	-	-	-	-	-	-	
LD4	MLDJJ2-1-3-2	-	-	-	-	-	-	
LD5	MLDJJ2-1-3-3	-	-	-	-	-	-	
LD6	MLDJJ2-1-3-4	-		+	-	-	-	1
LD7	MLDHH0-1-4-1	-	-	-	-	-	-	
LD8	MLDHH0-1-4-2	-	-	-	+	-	-	1
LD9	MLDHH0-1-4-3	-	+	-	-	-	-	1
LD10	MLDHH0-1-4-4	-	-	-	-	-	-	
LD11	MLDHH0-1-4-5	-	+	-	+	-	-	2
LD12	MLDLL2-1-5-1	-	-	-	-	-	-	
LD13	MLDLL2-1-5-2	-	-	+	-	-	-	1
LD14	MLDLL2-1-5-3	+		+	+	-	-	3
LD15	MLDLL2-1-5-4	-		-	-	-	-	
LD16	MLDLL2-1-5-5	-		-	+	-	-	1
LD17	MLDLL2-1-5-6	-		-	-	-	-	
LD18	MLDRR1-1-6-1	-		+	+	-	-	2
LD19	MLDRR1-1-6-2	-		-	+	-	-	1
LD20	MLDRR1-1-6-3	-		-	-	-	-	
LD21	MLDRR1-1-6-4	+		-		-	-	1
LD22	MLDRR1-1-6-5	-		-	+	-	-	1
LD23	MLDRR1-1-6-6	-		-	-	-	-	
LD24	MLDRR1-1-6-7	+		-	+	-	-	2

A ‘+’ indicates the presence; A ‘-‘ indicates the absence of disease; A blank cell indicates ‘not determined.’ Animal IDs with a ‘C’ belong to Control group, those with a ‘D’ belong to DDT group and those with a ‘LD’ belong to lower dose DDT group. See ‘Materials and Methods’ section for disease assessment in rats. The number of animals per litter (litter representation) mean ± SEM used for each specific disease/abnormality assessment within the control, DDT or lower dose DDT lineages were not found to be statistically different ($p>0.05$), so no litter bias detected.

Supplemental Table S2B.
Individual disease incidence in F1 generation male rats of Control, DDT and Lower Dose DDT lineages.

Serial Number	Rat ID	Puberty	Testis	Prostate	Kidney	Obesity	Tumor	Total Disease
C1	MCTT2-1-1-6	-	-	+	-	-	-	1
C2	MCTT2-1-1-7	-	+	-	+	-	-	2
C3	MCTT2-1-1-8	-	+	-	-	-	-	1
C4	MCTT2-1-1-9	-	-	-	-	-	-	
C5	MCWW0-1-2-10	-	-	-	-	-	-	
C6	MCWW0-1-2-11	-		-	-	-	-	
C7	MCAA0-1-3-6	-	-	-	+	-	-	1
C8	MCAA0-1-3-7	-	-	+	-	-	-	1
C9	MCAA0-1-3-8	-	-	-	+	-	-	1
C10	MCAA0-1-3-9	-	-	-	+	-	-	1
C11	MCGG1-1-4-5	-	-	+	-	-	-	1
C12	MCGG1-1-4-6	-	-	-	-	-	-	
C13	MCGG1-1-4-7	-	-	-	+	-	-	1
C14	MCGG1-1-4-8	-	-	-	-	-	-	
C15	MCWW0-1-6-7	-	-	+	-	+	-	2
C16	MCWW0-1-6-8	-	-	-	-	-	-	
C17	MCWW0-1-6-9	-	-	-	-	-	-	
C18	MCWW0-1-6-10	-	-	-	-	-	-	
C19	MCWW0-1-6-11	-			-	-	-	
C20	MCWW0-1-6-12	-	-	+	-	-	-	1
C21	MCGG2-1-7-6	-	-	-	-	-	-	
C22	MCGG2-1-7-7	-	-	-	-	-	-	
C23	MCGG2-1-7-8	-	-	-	-	-	-	
C24	MCGG2-1-7-9	-	-	-	-	-	-	
C25	MCZZ0-1-8-6	-	-	-	-	-	-	
C26	MCZZ0-1-8-7	-	-	-	-	-	-	
C27	MCZZ0-1-8-8	-	-	-	-	+	-	1
C28	MCZZ0-1-8-9	-	+	-	-	+	-	2
C29	MCZZ0-1-8-10	+	-	+	-	-	-	2

Serial Number	Rat ID	Puberty	Testis	Prostate	Kidney	Obesity	Tumor	Total Disease
D1	MDLL2-1-1-5	-	-	+	+	-	-	2
D2	MDLL2-1-1-6	-	-	-	-	-	+	1
D3	MDAA1-1-2-6	-	-	+	-	-	-	1
D4	MDAA1-1-2-7	-	-	-	+	-	-	1
D5	MDAA1-1-2-8	-	-	-	-	-	-	
D6	MDAA1-1-2-9	-	-	+	-	-	-	1
D7	MDAA1-1-2-11	+	-			-	-	1
D8	MDGG0-1-3-10	-	-	-	-	-	-	
D9	MDGG0-1-3-11	-	-	+	-	-	+	2
D10	MDGG0-1-3-12	-	-	+	-	-	+	2

Serial Number	Rat ID	Puberty	Testis	Prostate	Kidney	Obesity	Tumor	Total Disease
LD1	MLDLL0-1-1-2	-	-	-	+	-	-	1
LD2	MLDLL0-1-1-3	-	-	+	+	-	-	2
LD3	MLDLL0-1-1-4	-	-	+	+	-	-	2
LD4	MLDLL0-1-1-7	-	-		+	+	-	2
LD5	MLDJJ2-1-3-5	-	-	+	+	+	-	3
LD6	MLDJJ2-1-3-6	-	+	+	-	+	-	3
LD7	MLDJJ2-1-3-7	-	-	-	+	-	-	1
LD8	MLDJJ2-1-3-8	-	-	-	-	+	-	1
LD9	MLDJJ2-1-3-9	-	-	+	-	-	+	2
LD10	MLDHH0-1-4-6	+	-	-	+	-	-	2
LD11	MLDHH0-1-4-7	+	-	+	-	-	-	2
LD12	MLDHH0-1-4-8	+	-	+	-	-	-	2
LD13	MLDHH0-1-4-9	+	-	-	+	-	-	2
LD14	MLDLL2-1-5-7	-			-	-	+	1
LD15	MLDLL2-1-5-8	-	-	-	+	-	-	1
LD16	MLDLL2-1-5-9	-	-	+	-	-	-	1
LD17	MLDLL2-1-5-10	-	-	-	+	-	-	1
LD18	MLDRR1-1-6-8	-	-	+	+	-	-	2
LD19	MLDRR1-1-6-9	-	-	+	+	-	-	2
LD20	MLDRR1-1-6-10	-	-	-	-	-	-	
LD21	MLDRR1-1-6-11	-	+	+	+	-	-	3
LD22	MLDRR1-1-6-12	-	+	-	+	-	-	2

A '+' indicates the presence; A '-' indicates the absence of disease; A blank cell indicates 'not determined.' Animal IDs with a 'C' belong to Control group, those with a 'D' belong to DDT group and those with a 'LD' belong to lower dose DDT group. See 'Materials and Methods' section for disease assessment in rats. The number of animals per litter (litter representation) mean ± SEM used for each specific disease/abnormality assessment within the control, DDT or lower dose DDT lineages were not found to be statistically different ($p>0.05$), so no litter bias detected.
