# Supplementary material for: Ancestral dichlorodiphenyltrichloroethane (DDT) exposure promotes epigenetic transgenerational inheritance of obesity
Source: BMC Med. 2013 Oct 23;11:228. doi: 10.1186/1741-7015-11-228 (PMC3853586; doi:10.1186/1741-7015-11-228)
Supplement: Additional file 5: Table S3 — (A) Individual disease incidence in F3 generation female rats of control, dichlorodiphenyltrichloroethane (DDT) and lower dose DDT lineages. (B) Individual disease incidence in F3 generation male rats of control, DDT and lower dose DDT lineages. ?+? indicates the presence and ?-? indicates the absence of disease; a blank cell indicates ?not determined?. Animal IDs with a ?C? belong to the control group, those with a ?D? belong to the DDT group and those with ?LD? belong to the lower dose DDT group. See Methods section for disease assessment in rats. The number of animals per litter (litter representation) mean???SEM used for each specific disease/abnormality assessment within the control, DDT or lower dose DDT lineages were not found to be statistically different (P >0.05), so no litter bias was detected. [file 1741-7015-11-228-S5.pdf]

Supplemental Table S3A

A. Individual disease incidence in F3 generation female rats of Control, DDT and Lower Dose DDT lineages.

Serial #	Rat ID	Puberty	Ovary	Uterus	Kidney	Tumor	Obesity	Total Disease
C1	MCTT2-3-1-1	-	-	-	+	-	-	1
C2	MCTT2-3-1-2	-	-	+	-	-	-	1
C3	MCGG1-3-2-1	+	-	-	-	-	-	1
C4	MCGG1-3-2-2	+	-	+	-	-	-	2
C5	MCGG1-3-3-1	-	-	-	-	-	-	
C6	MCGG1-3-3-2	-	-	-	-	-	-	
C7	MCWW0-3-4-1	-	-	-	-	-	-	
C8	MCWW0-3-4-2	-	-	-	-	-	-	
C9	MCGG1-3-6-1	-	-	-	-	-	-	
C10	MCAA0-3-7-1	-	-	-	-	-	-	
C11	MCAA0-3-7-2	-		-	-	-	+	1
C12	MCGG2-3-8-1	-	-	-	+	-	-	1
C13	MCGG2-3-8-2	-		-	+	-	-	1
C14	MCTT2-3-9-1	-	-	-	+	-	-	1
C15	MCTT2-3-9-2	-		+	+	-	-	2
C16	MCWW0-3-10-1	-	-	-	-	-	-	
C17	MCGG2-3-12-1	-		-	-	-	-	
C18	MCGG1-3-13-1	-		-	-	-	-	
C19	MCGG1-3-14-1	-		+	-	-	-	1
C20	MCGG1-3-15-1	-		-	-	-	-	
C21	MCWW0-3-16-1	-	-	-	-	-	-	
C22	MCWW0-3-16-2	-		-	-	-	-	
C23	MCGG1-3-17-1	-	-	-	-	-	-	
C24	MCAA0-3-18-1	-	-	-	-	-	-	
C25	MCAA0-3-19-1	-	-	+	-	-	-	1
C26	MCAA0-3-19-2	-	-	+	-	-	-	1
C27	MCGG2-3-20-1	-	-	-	-	-	-	
C28	MCAA0-3-21-1	-	-	-	-	-	-	
C29	MCGG1-3-22-1	-		-	-	-	-	

Serial #	Rat ID	Puberty	Ovary	Uterus	Kidney	Tumor	Obesity	Total Disease
D1	MDGG0-3-1-1	-	-	-	+	-	+	2
D2	MDGG0-3-1-2	-	+	+	+	-	-	3
D3	MDGG0-3-1-3	-	-	+	-	-	-	1
D4	MDGG0-3-2-1	-	-	+	+	-	-	2
D5	MDGG0-3-2-2	-	+	-	+	-	+	3
D6	MDGG0-3-2-3	-	+	-	-	-	-	1
D7	MDGG0-3-3-1	+	+	+	-	-	-	3
D8	MDGG0-3-3-2	-	+	+	+	-	-	3
D9	MDGG0-3-3-3	-	-	-	+	-	+	2
D10	MDAA1-3-4-2	-	-	+	-	-	-	1
D11	MDAA1-3-4-3	-		+	+	-	-	2

D12	MDGG0-3-5-1	-		+		-	-	1
D13	MDGG0-3-5-2	-	+	-	-	-	-	1
D14	MDGG0-3-5-3	-		-	-	-	-	
D15	MDGG0-3-6-1	-	+	-	-	-	-	1
D16	MDAA1-3-7-1	-	+	+	+	-	-	3
D17	MDGG0-3-8-1	-	+	+	-	-	-	2
D18	MDGG0-3-8-2	-	-	+	-	-	-	1
D19	MDAA1-3-9-2	+	+	+	-	-	-	3
D20	MDAA1-3-9-6	-		+		-	-	1
D21	MDAA1-3-10-1	-	+	+	-	-	-	2
D22	MDAA1-3-10-2	-	-	-	-	-	-	
D23	MDGG0-3-11-1	+	-	+	-	-	-	2
D24	MDGG0-3-11-2	+	+	+	-	-	-	3
D25	MDGG0-3-12-1	-	+	+			+	3
D26	MDGG0-3-12-2	-		+				1
D27	MDGG0-3-12-3	-		-		+		1
D28	MDGG0-3-13-1	-		+				1
D29	MDGG0-3-13-2	-		+				1
D30	MDGG0-3-13-3	-		+				1
D31	MDGG0-3-13-5	-		+				1

Serial #	Rat ID	Puberty	Ovary	Uterus	Kidney	Tumor	Obesity	Total Disease
LD1	MLDLL2-3-1-1	-	+	-	-	-	+	2
LD2	MLDLL2-3-1-2	-	-	-	-	-	-	
LD3	MLDLL2-3-2-1	-	+	-	-	-	+	2
LD4	MLDLL2-3-2-2	-	+	-	+	-	-	2
LD5	MLDRR1-3-3-1	-	-	-	+	-	-	1
LD6	MLDRR1-3-3-2	-	+	-	+	-	-	2
LD7	MLDLL2-3-4-1	-	-	-	-	-	+	1
LD8	MLDKK0-3-5-1	-	-	-	+	-	+	2
LD9	MLDKK0-3-5-2	-	-	-	-	-	+	1
LD10	MLDHH0-3-6-1	-	-	-	+	-	+	2
LD11	MLDHH0-3-6-2	-	-	+	+	-	+	3
LD12	MLDJJ2-3-7-1	+	+	-	-	-	+	3
LD13	MLDJJ2-3-7-2	-		-	-	-	-	
LD14	MLDJJ2-3-8-1	-	-	-	-	-	-	
LD15	MLDJJ2-3-8-2	-	-	-	+	-	-	1
LD16	MLDJJ2-3-9-1	-		-	-	-	-	
LD17	MLDJJ2-3-9-2	-	-	-	-	-	-	
LD18	MLDKK0-3-10-1	-	-	-	-	-	-	
LD19	MLDJJ2-3-11-1	+	-	-	-	-	+	2
LD20	MLDJJ2-3-11-5	-		-		-	+	1
LD21	MLDJJ2-3-12-1	-		+	+	-	-	2
LD22	MLDJJ2-3-12-2	-		-	+	-	+	2
LD23	MLDJJ2-3-13-1	+		-	-	-	+	2
LD24	MLDJJ2-3-14-1	-	-	-	+	-	+	2
LD25	MLDJJ2-3-14-3	-	+	-	-	-	+	2
LD26	MLDRR1-3-15-1	-	+	-	-	-	-	1

LD27	MLDRR1-3-15-2	-		-	+	-	-	1
LD28	MLDHH0-3-16-1	-		+	+	-	-	2
LD29	MLDHH0-3-16-2	-		-	+	-	-	1
LD30	MLDLL2-3-17-1	-		-	+	-	-	1
LD31	MLDRR1-3-18-1	+		-	-	-	+	2
LD32	MLDRR1-3-19-1	-		-	+	-	+	2

A ‘+’ indicates the presence; A ‘-‘ indicates the absence of disease; A blank cell indicates ‘not determined.’ Animal IDs with a ‘C’ belong to Control group, those with a ‘D’ belong to DDT group and those with a ‘LD’ belong to lower dose DDT group. See ‘Materials and Methods’ section for disease assessment in rats. The number of animals per litter (litter representation) mean ± SEM used for each specific disease/abnormality assessment within the control, DDT or lower dose DDT lineages were not found to be statistically different (p>0.05), so no litter bias detected.

Supplemental Table S3B.

Individual disease incidence in F3 generation male rats of Control, DDT and Lower Dose DDT lineages.

Serial #	Rat ID	Puberty	Testis	Prostate	Kidney	Tumor	Obesity	Total Disease
C1	MCTT2-3-1-5	-	-	-	-	-	-	
C2	MCTT2-3-1-6	-	-	-	-	-	-	
C3	MCGG1-3-2-10	-	-	-	-	-	-	
C4	MCGG1-3-2-11	-	-	-	-	-	-	
C5	MCGG1-3-3-6	-	+	-	-	-	-	1
C6	MCGG1-3-3-7	-	+	-	-	-	-	1
C7	MCWW0-3-4-4	-	-	+	+	-	+	3
C8	MCWW0-3-4-5	-	-	-	-	-	-	
C9	MCGG1-3-6-8	-	-	+	+	-	-	2
C10	MCAA0-3-7-6	-	-	-	-	-	-	
C11	MCAA0-3-7-7	-	-	-	-	-	-	
C12	MCGG2-3-8-7	-	+	-	-	-	-	1
C13	MCGG2-3-8-8	-	-	-	-	-	-	
C14	MCTT2-3-9-5	-	-	-	-	-	+	1
C15	MCTT2-3-9-6	-	-	-	-	-	+	1
C16	MCWW0-3-10-9	-	+	-	-	-	-	1
C17	MCWW0-3-10-10	-	-	-	-	-	+	1
C18	MCGG2-3-12-4	-	-	-	-	-	+	1
C19	MCGG1-3-13-5	-	-	+	-	-	-	1
C20	MCGG1-3-14-4	-	-	-	-	-	-	
C21	MCGG1-3-15-3	-	+	-	-	-	-	1
C22	MCWW0-3-16-5	-	-	+	-	-	-	1
C23	MCWW0-3-16-6	-	-	-	-	-	-	
C24	MCGG1-3-17-4	+	-	-	-	-	-	1
C25	MCAA0-18-2	-	-	-	-	-	-	
C26	MCAA0-3-19-5	-	-	-	-	-	+	1
C27	MCAA0-3-19-6	-	+	-	+	-	-	2
C28	MCGG2-3-20-9	-	-	-	-	-	+	1
C29	MCGG2-3-20-10	-				+	-	1
C30	MCAA0-3-21-5	-	-	+	-	-	-	1
C31	MCGG1-3-22-8	-	-	-	-	-	-	

Serial #	Rat ID	Puberty	Testis	Prostate	Kidney	Tumor	Obesity	Total Disease
D1	MDGG0-3-1-8	-	-	-	-	-	+	1
D2	MDGG0-3-1-9	-	+	-	+	-	-	2
D3	MDGG0-3-1-10	-	-	-	-	-	-	
D4	MDGG0-3-2-10	-	-	-	+	-	-	1
D5	MDGG0-3-2-11	-	-	-	+	-	-	1
D6	MDGG0-3-2-12	-	-	-	-	-	-	
D7	MDGG0-3-3-12	-	-	+	-	-	-	1
D8	MDGG0-3-3-13	-	-	-	-	-	+	1
D9	MDGG0-3-3-14	-	-	-	-	-	+	1

D10	MDAA1-3-4-7	-	+	+	-	-	+	3
D11	MDAA1-3-4-8	-	-	-	+	-	+	2
D12	MDAA1-3-4-9	-	-	-	-	-	+	1
D13	MDGG0-3-5-10	-	+	-	-	-	+	2
D14	MDGG0-3-5-11	-	+	-	-	-	+	2
D15	MDGG0-3-5-12	-	-	+	-	-	-	1
D16	MDGG0-3-6-7	-	-	-	-	-	+	1
D17	MDAA1-3-7-4	-	+	-	-	-	+	2
D18	MDGG0-3-8-7	-	+	-	+	-	+	3
D19	MDGG0-3-8-8	-	+	-	-	-	+	2
D20	MDAA1-3-9-8	-	-	+	-	-	+	2
D21	MDAA1-3-9-9	-	+	-	-	-	+	2
D22	MDAA1-3-10-4	+	+	-	+	-	+	4
D23	MDAA1-3-10-5	+	+	+	-	-	+	4
D24	MDGG0-3-11-6	-	+	-	+	-	+	3
D25	MDGG0-3-11-7	-	+	-	+	-	+	3
D26	MDGG0-3-12-5	-	-	+	+	-	+	3
D27	MDGG0-3-12-6	-	+	-	+	-	+	3
D28	MDGG0-3-13-12	-	+	-	-	-	+	2
D29	MDGG0-3-13-13	-	-	-	-	-	+	1
D30	MDGG0-3-13-14	-				-	+	1

Serial #	Rat ID	Puberty	Testis	Prostate	Kidney	Tumor	Obesity	Total Disease
LD1	MLDLL2-3-1-6	-	-	-	+	-	+	2
LD2	MLDLL2-3-1-7	-	-	+	+	-	+	3
LD3	MLDLL2-3-1-8	+	+			-	+	3
LD4	MLDLL2-3-2-6	-	-	-	-	-	+	1
LD5	MLDLL2-3-2-7	-	-	+	+	-	+	3
LD6	MLDRR1-3-3-4	-	+	-	-	-	-	1
LD7	MLDRR1-3-3-5	-	-	-	-	-	-	
LD8	MLDRR1-3-3-6	-	-	-	-	-	-	
LD9	MLDLL2-3-4-3	-	-	+	-	-	-	1
LD10	MLDKK0-3-5-6	-	+	-	-	-	+	2
LD11	MLDKK0-3-5-7	-	-	-	+	-	+	2
LD12	MLDHH0-3-6-9	-	+	-	-	-	+	2
LD13	MLDHH0-3-6-10	-	-	-	-	-	+	1
LD14	MLDJJ2-3-7-6	-	-	-	+	-	-	1
LD15	MLDJJ2-3-7-7	-	-	+	+	-	-	2
LD16	MLDJJ2-3-8-12	-	-	+	+	-	+	3
LD17	MLDJJ2-3-8-13	-	-	+	+	-	-	2
LD18	MLDJJ2-3-9-8	-	-	-	+	-	+	2
LD19	MLDJJ2-3-9-9	-	-	-	+	-	+	2
LD20	MLDKK0-3-10-9	-	-	+	-	-	+	2
LD21	MLDJJ2-3-11-5	-	-	-	+	-	-	1
LD22	MLDJJ2-3-12-2	-	-	-	+	-	-	1
LD23	MLDJJ2-3-14-9	-	+	-	+	-	-	2
LD24	MLDJJ2-3-14-10	-	+	+	+	-	-	3

LD25	MLDRR1-3-15-10	-	+	-	+	-	-	2
LD26	MLDRR1-3-15-11	-	-	+	-	-	-	1
LD27	MLDHH0-3-16-7	-	-	-	+	-	+	2
LD28	MLDLL2-3-17-7	-	+	-	-	-	+	2
LD29	MLDRR1-3-18-9	-	-	-	+	-	+	2
LD30	MLDRR1-3-19-5	-	+	-	+	-	-	2

A '+' indicates the presence; A '-' indicates the absence of disease; A blank cell indicates 'not determined.' Animal IDs with a 'C' belong to Control group, those with a 'D' belong to DDT group and those with a 'LD' belong to lower dose DDT group. See 'Materials and Methods' section for disease assessment in rats. The number of animals per litter (litter representation) mean ± SEM used for each specific disease/abnormality assessment within the control, DDT or lower dose DDT lineages were not found to be statistically different ($p>0.05$), so no litter bias detected.
