# Supplementary material for: Ancestral dichlorodiphenyltrichloroethane (DDT) exposure promotes epigenetic transgenerational inheritance of obesity
Source: BMC Med. 2013 Oct 23;11:228. doi: 10.1186/1741-7015-11-228 (PMC3853586; doi:10.1186/1741-7015-11-228)
Supplement: Additional file 6: Table S4 — (A) Individual disease incidence in F4 generation outcross female rats of control and lower dose dichlorodiphenyltrichloroethane (DDT) lineages. (B) Individual disease incidence in F4 generation outcross male rats of control and lower dose DDT lineages. ?+? indicates the presence and ?-? indicates the absence of disease; a blank cell indicates ?not determined?. Animal IDs with a ?C? belong to the control group, those with a ?LD? belong to the lower dose DDT group. See Methods section for disease assessment in rats. The number of animals per litter (litter representation) mean???SEM used for each specific disease/abnormality assessment within the control or lower dose DDT lineages were not found to be statistically different (P >0.05), so no litter bias was detected. [file 1741-7015-11-228-S6.pdf]

Supplemental Table S4A.

Individual disease incidence in F4 generation Outcross female rats of Control and Lower Dose DDT lineages.

Serial Number	Rat ID	Puberty	Ovary	Uterus	Kidney	Tumor	Obesity	Total Disease
C1	MOC5-O4-1-1	-	-	-	-	-	-	
C2	MOC5-O4-1-2	-	-	-	-	-	-	
C3	MOC5-O4-1-3	-	-	-	-	-	-	
C4	MOC5-O4-1-4	-	-	-	+	-	-	1
C5	MOC5-O4-1-5	+	-	-	+	-	-	2
C6	MOC5-O4-1-6	+	-	-	-	-	-	1
C7	MOC5-O4-1-7	-	-	-	+	-	-	1
C8	MOC4-O4-2-1	-	-	-	-	-	-	
C9	MOC4-O4-2-2	-	-	-	-	-	+	1
C10	MOC4-O4-2-3	-	-	-	-	-	-	
C11	MOC4-O4-2-4	-	+	-	+	-	-	2
C12	MOC4-O4-2-6	-		-		-	+	1
C13	MOC4-O4-2-7	-		-		-	+	1
C14	MOC7-04-3-1	-		-		+	-	1
C15	MOC7-04-3-3	-		+		-	-	1
C16	MOC8-O4-4-1	-	-	-	-	-	-	
C17	MOC8-O4-4-2	-	-	-	-	-	-	
C18	MOC2-O4-5-1	-	+	-	-	-	-	1
C19	MOC2-O4-5-2	-		-		-	+	1
C20	MOC2-O4-5-3	-		-		-	+	1
C21	MOC2-O4-5-6	-		-		-	+	1
C22	MOC1-04-6-1	-		+		-	-	1
C23	MOC1-04-6-2	-		+		-	-	1
C24	MOC6-O4-7-1	-	-	-	-	-	+	1
C25	MOC6-O4-7-3	-		-		-	+	1

Serial Number	Rat ID	Puberty	Ovary	Uterus	Kidney	Tumor	Obesity	Total Disease
LD1	MOLD9-O4-1-1	-	+	-	-	-	-	1
LD2	MOLD9-O4-1-2	-	-	-	-	-	-	
LD3	MOLD9-O4-1-3	-	-	-	-	-	-	
LD4	MOLD9-O4-1-4	-	-	-	+	-	-	1
LD5	MOLD9-O4-1-5	-	-	+	-	-	-	1
LD6	MOLD2-O4-2-1	-	-	-	-	-	+	1
LD7	MOLD2-O4-2-2	-	-	-	-	-	+	1
LD8	MOLD2-O4-2-3	-		-		-	+	1
LD9	MOLD2-O4-2-4	-		-		-	+	1
LD10	MOLD2-O4-2-5	-		-		-	+	1
LD11	MOLD3-O4-3-1	-	+	-	-	-	-	1
LD12	MOLD3-O4-3-2	-	+	-	+	-	-	2
LD13	MOLD3-O4-3-3	-	-	-	+	-	-	1
LD14	MOLD3-O4-3-4	-	-	-	-	-	+	1
LD15	MOLD3-O4-3-5	-	+	-	+	-	-	2

LD16	MOLD1-O4-4-1	-	-	-	+	-	+	2
LD17	MOLD1-O4-4-2	-	-	-	-	-	-	
LD18	MOLD1-O4-4-3	-	-	-	-	-	+	1
LD19	MOLD1-O4-4-4	-		-		-	+	1
LD20	MOLD-04-5-2	-		-		-	+	1
LD21	MOLD-04-6-1	-		-		-	+	1
LD22	MOLD-04-6-4	-		-		-	+	1

A '+' indicates the presence; A '-' indicates the absence of disease; A blank cell indicates 'not determined.' Animal IDs with a 'C' belong to Control group, those with a 'LD' belong to lower dose DDT group. See 'Materials and Methods' section for disease assessment in rats. The number of animals per litter (litter representation) mean ± SEM used for each specific disease/abnormality assessment within the control or lower dose DDT lineages were not found to be statistically different ($p>0.05$), so no litter bias detected.

Supplemental Table S4B.

Individual disease incidence in F4 generation Outcross male rats of Control and Lower Dose DDT lineages.

Serial Number	Rat ID	Puberty	Testis	Kidney	Tumor	Obesity	Total Disease
C1	MOC5-O4-1-9	-	-	-	-	-	
C2	MOC5-O4-1-10	-	+	+	-	-	2
C3	MOC5-O4-1-11	-	-	-	-	+	1
C4	MOC5-O4-1-12	-	-	+	-	-	1
C5	MOC4-O4-2-9	-	-	-	-	-	
C6	MOC4-O4-2-10	-	-	-	-	-	
C7	MOC4-O4-2-11	-	-	-	-	-	
C8	MOC4-O4-2-12	-	-	-	-	-	
C9	MOC4-O4-2-13	-	+	-	-	-	1
C10	MOC7-O4-3-6	-	-	+	-	+	2
C11	MOC7-O4-3-7	-		+	-	-	1
C12	MOC7-O4-3-8	-			-	+	1
C13	MOC7-O4-3-9	-		+	-	-	1
C14	MOC7-O4-3-11	-			-	+	1
C15	MOC8-04-4-5	-			-	+	1
C16	MOC8-04-4-6	-			-	+	1
C17	MOC2-04-5-10	-		+	-	-	1
C18	MOC2-04-5-11	-		+	-	-	1
C19	MOC1-04-6-5	-		+	-	+	2
C20	MOC1-04-6-6	-		+	-	-	1
C21	MOC1-04-6-7	-		+	-	-	1
C22	MOC1-04-6-8	-			-	+	1
C23	MOC1-04-6-9	-			-	+	1
C24	MOC1-04-6-10	-			-	+	1
C25	MOC6-O4-7-6	-	-	+	-	-	1
C26	MOC6-O4-7-7	-	-	-	-	+	1
C27	MOC6-O4-7-8	-	-	-	-	+	1
C28	MOC6-O4-7-9	-	-	+	-	-	1
C29	MOC6-O4-7-10	-	-	-	-	-	

Serial Number	Rat ID	Puberty	Testis	Kidney	Tumor	Obesity	Total Disease
LD1	MOLD9-O4-1-9	-	+	-	-	+	2
LD2	MOLD9-O4-1-10	-	-	+	-	+	2
LD3	MOLD9-O4-1-11	-	-	+	-	-	1
LD4	MOLD2-O4-2-8	-	-	+	-	+	2
LD5	MOLD2-O4-2-9	-	-	+	-	-	1
LD6	MOLD2-O4-2-10	-	-	+	-	+	2
LD7	MOLD2-O4-2-11	-	-	+	-	+	2
LD8	MOLD2-O4-2-12	-	-	+	-	-	1
LD9	MOLD3-O4-3-8	-	-	+	-	-	1
LD10	MOLD3-O4-3-9	-	-	+	-	-	1
LD11	MOLD3-O4-3-10	-	+	+	-	-	2

LD12	MOLD1-O4-4-7	-	+	+	-	-	2
LD13	MOLD1-O4-4-8	-	+	+	-	+	3
LD14	MOLD1-O4-4-9	-	-	-	-	+	1
LD15	MOLD8-O4-5-6	-	-	+	-	+	2
LD16	MOLD8-O4-5-7	-			-	+	1
LD17	MOLD8-O4-5-8	-			-	+	1

A ‘+’ indicates the presence; A ‘-‘ indicates the absence of disease; A blank cell indicates ‘not determined.’ Animal IDs with a ‘C’ belong to Control group, those with a ‘LD’ belong to lower dose DDT group. See ‘Materials and Methods’ section for disease assessment in rats. The number of animals per litter (litter representation) mean ± SEM used for each specific disease/abnormality assessment within the control or lower dose DDT lineages were not found to be statistically different ($p>0.05$), so no litter bias detected.
