# Supplementary material for: Ancestral dichlorodiphenyltrichloroethane (DDT) exposure promotes epigenetic transgenerational inheritance of obesity
Source: BMC Med. 2013 Oct 23;11:228. doi: 10.1186/1741-7015-11-228 (PMC3853586; doi:10.1186/1741-7015-11-228)
Supplement: Additional file 7: Table S5 — (A) Individual disease incidence in F4 generation reverse outcross female rats of control and lower dose dichlorodiphenyltrichloroethane (DDT) lineages. (B) Individual disease incidence in F4 generation Reverse Outcross male rats of Control and Lower Dose DDT lineages. ?+? indicates the presence and ?-? indicates the absence of disease; a blank cell indicates ?not determined?. Animal IDs with a ?C? belong to the control group, those with a ?LD? belong to the lower dose DDT group. See Methods section for disease assessment in rats. The number of animals per litter (litter representation) mean???SEM used for each specific disease/abnormality assessment within the control or lower dose DDT lineages were not found to be statistically different (P >0.05), so no litter bias was detected. [file 1741-7015-11-228-S7.pdf]

Supplemental Table S5A.

Individual disease incidence in F4 generation Reverse Outcross female rats of Control and Lower Dose DDT lineages.

Serial Number	Rat ID	Puberty	Ovary	Uterus	Kidney	Tumor	Obesity	Total Disease
C1	MCCGG1-R4-1-1	-	-	-	-	-	-	
C2	MCCGG1-R4-1-2	-	-	-	-	-	-	
C3	MCCGG1-R4-1-3	-	-	+	-	-	-	1
C4	MCTT2-R4-2-1	-	-	+	-	-	+	2
C5	MCTT2-R4-2-2	-	-	+	-	-	+	2
C6	MCTT2-R4-2-4	-		+		-	-	1
C7	MCTT2-R4-2-5	-		+		-	-	1
C8	MCWW0-R4-3-1	-	+	-		-	+	2
C9	MCWW0-R4-3-2	-		+		-	+	2
C10	MCGG1-R4-4-1	+	-	-	-	-	-	1
C11	MCGG1-R4-4-2	+	-	-	-	-	-	1
C12	MCGG1-R4-4-3	-	-	+	-	-	-	1
C13	MCAA0-R4-5-1	-	-	-	-	-	-	
C14	MCAA0-R4-5-2	-	-	-	-	-	-	
C15	MCGG1-R4-6-1	-	-	+	+	-	-	2
C16	MCGG1-R4-6-2	-	-	-	-	-	-	
C17	MCGG1-R4-6-3	-	-	-	-	-	-	
C18	MCGG2-R4-7-1	-	-	-	+	-	-	1
C19	MCGG2-R4-7-2	-	-	-	+	-	-	1

Serial Number	Rat ID	Puberty	Ovary	Uterus	Kidney	Tumor	Obesity	Total Disease
LD1	MLDLL2-R4-2-1	-	-	-	+	-	-	1
LD2	MLDLL2-R4-2-2	-	-	-	-	-	-	
LD3	MLDLL2-R4-2-3	-	+	-	-	-	-	1
LD4	MLDLL2-R4-2-4	-	+	-	-	-	-	1
LD5	MLDRR1-R4-3-1	-	+	-	+	-	-	2
LD6	MLDRR1-R4-3-2	-	+	-	+	-	-	2
LD7	MLDHH0-R4-4-1	-	+	+		-	-	2
LD8	MLDHH0-R4-4-2	-		+		-	-	1
LD9	MLDJJ2-R4-5-1	-	-	-	+	-	-	1
LD10	MLDJJ2-R4-5-2	-	+	-	-	-	-	1
LD11	MLDJJ2-R4-5-3	-	-	-	-	-	-	
LD12	MLDJJ2-R4-5-4	-	-	-	-	-	-	
LD13	MLDJJ2-R4-5-5	-	-	-	+	-	-	1
LD14	MLDKK0-R4-6-3	-		-		-	+	1
LD15	MLDJJ2-R4-7-1	-	+	+	+	-	-	3
LD16	MLDLL2-R4-8-1	-	-	-	-	-	+	1
LD17	MLDLL2-R4-8-2	-	+	-	+	-	+	3
LD18	MLDLL2-R4-8-3	-		-		-	+	1
LD19	MLDLL2-R4-8-4	-		-		-	+	1

A '+' indicates the presence; A '-' indicates the absence of disease; A blank cell indicates 'not determined.' Animal IDs with a 'C' belong to Control group, those

with a ‘LD’ belong to lower dose DDT group. See ‘Materials and Methods’ section for disease assessment in rats. The number of animals per litter (litter representation) mean ± SEM used for each specific disease/abnormality assessment within the control or lower dose DDT lineages were not found to be statistically different ($p>0.05$), so no litter bias detected.

Supplemental Table S5B.

Individual disease incidence in F4 generation Reverse Outcross male rats of Control and Lower Dose DDT lineages.

Serial Number	Rat ID	Puberty	Testis	Kidney	Tumor	Obesity	Total Disease
C1	MCTT2-R4-2-9	-		+	-	-	1
C2	MCWW0-R4-3-4	-	-	-	-	-	
C3	MCWW0-R4-3-5	-	-	-	-	-	
C4	MCWW0-R4-3-6	-	-	+	-	-	1
C5	MCWW0-R4-3-7	-	-	-	-	-	
C6	MCWW0-R4-3-8	-	+	-	-	-	1
C7	MCWW0-R4-3-9	-	-	-	-	-	
C8	MCGG1-R4-4-6	-	-	-	-	-	
C9	MCGG1-R4-4-7	-	-		-	-	
C10	MCGG1-R4-4-8	-	-	-	-	-	
C11	MCGG1-R4-4-10	-	-	-	-	-	
C12	MCGG1-R4-4-11	-	-	-	-	-	
C13	MCGG1-R4-4-12	-	-	+	-	-	1
C14	MCGG1-R4-6-8	-		+	-	-	1
C15	MCGG1-R4-6-9	-			-	+	1
C16	MCGG2-R4-7-4	-		+	-	-	1
C17	MCGG2-R4-7-6	-	-	+	-	-	1
C18	MCGG2-R4-7-7	-	-	+	-	-	1
C19	MCGG2-R4-7-8	-	-	+	-	-	1

Serial Number	Rat ID	Puberty	Testis	Kidney	Tumor	Obesity	Total Disease
LD1	MLDLL2-R4-1-2	-	-	+	-	-	1
LD2	MLDLL2-R4-2-8	-	+	-	-	+	2
LD3	MLDLL2-R4-2-9	-	+	+	-	-	2
LD4	MLDRR1-R4-3-5	-			-	+	1
LD5	MLDRR1-R4-3-7	-	-	+	-	-	1
LD6	MLDRR1-R4-3-9	-	-	+	-	-	1
LD7	MLDRR1-R4-3-10	-	-	+	-	+	2
LD8	MLDHH0-R4-4-5	-	-	+	-	+	2
LD9	MLDJJ2-R4-5-8	-	-	-	-	+	1
LD10	MLDJJ2-R4-5-9	-	+	+	-	-	2
LD11	MLDJJ2-R4-5-10	-	-	+	-	-	1
LD12	MLDKK0-R4-6-6	-			-	+	1
LD13	MLDKK0-R4-6-7	-	-	+	-	+	2
LD14	MLDKK0-R4-6-8	-	-	+	-	+	2
LD15	MLDJJ2-R4-7-2	-	+	-	-	+	2
LD16	MLDJJ2-R4-7-3	-	-	+	-	+	2
LD17	MLDJJ2-R4-7-4	-			-	+	1
LD18	MLDJJ2-R4-7-5	-			-	+	1
LD19	MLDLL2-R4-8-7	-	+	+	-	-	2
LD20	MLDLL2-R4-8-9	-			-	+	1
LD21	MLDLL2-R4-8-10	-			-	+	1

LD22	MLDLL2-R4-8-11	-			-	+	1

A ‘+’ indicates the presence; A ‘-‘ indicates the absence of disease; A blank cell indicates ‘not determined.’ Animal IDs with a ‘C’ belong to Control group, those with a ‘LD’ belong to lower dose DDT group. See ‘Materials and Methods’ section for disease assessment in rats. The number of animals per litter (litter representation) mean ± SEM used for each specific disease/abnormality assessment within the control or lower dose DDT lineages were not found to be statistically different ($p>0.05$), so no litter bias detected.
