# Supplementary material for: Ancestral dichlorodiphenyltrichloroethane (DDT) exposure promotes epigenetic transgenerational inheritance of obesity
Source: BMC Med. 2013 Oct 23;11:228. doi: 10.1186/1741-7015-11-228 (PMC3853586; doi:10.1186/1741-7015-11-228)
Supplement: Additional file 8: Table S6 — Dichlorodiphenyltrichloroethane (DDT) induced F3 generation sperm differential DNA methylation regions (DMR) (intersection). Epimutations found in F3-generation sperm after exposure of F0 generation gestating females to DDT, obtained by intersection of the five results of three methylated DNA fragment immunoprecipitation (MeDIP)-chip comparative hybridizations. The genes in bold were found to be unique DMR associated only with DDT-induced DMR. [file 1741-7015-11-228-S8.pdf]

Supplemental Table S6.

DDT induced F3 generation sperm differential DNA methylation regions (DMR) (Intersection)

Gene Symbol	Chr	Start	End	Gene ID	Min P Value	Gene Title
**Znf575**	**1**	**79870298**	**79872347**	**308430**	**3.69E-23**	**Zinc finger protein 575**
**Trpm4**	**1**	**95802352**	**95803340**	**171143**	**1.16E-09**	**Transient receptor potential cation channel**
Tsku	1	155621758	155622558	308843	7.75E-19	Tsukushin
**Sfxn3**	**1**	**250111583**	**250112379**	**65042**	**9.44E-08**	**Sideroflexin 3**
**Prkaa1**	**2**	**54327367**	**54328344**	**65248**	**1.83E-20**	**Protein kinase**
**Fxr1**	**2**	**120329766**	**120330481**	**361927**	**3.19E-14**	**Fragile X mental retardation**
**Bglap**	**2**	**180484821**	**180487344**	**25295**	**8.96E-32**	**Bone gamma-carboxyglutamate (gla) protein**
**Ppp6c**	**3**	**18997054**	**18997654**	**171121**	**3.92E-18**	**Protein phosphatase 6**
**Epc2**	**3**	**30420205**	**30420805**	**362132**	**4.26E-10**	**Enhancer of polycomb homolog 2 (Drosophila)**
**Meis2**	**3**	**102118424**	**102119024**	**311311**	**7.46E-13**	**Meis homeobox 2**
**Rps21**	**3**	**169311014**	**169311614**	**81775**	**4.56E-09**	**Ribosomal protein S21**
Clstn3	4	160664554	160666152	171393	7.21E-08	Calsyntenin 3
**Hcrtr1**	**5**	**149170407**	**149171007**	**25593**	**1.51E-09**	**Hypocretin (orexin) receptor 1**
Npc2	6	108815006	108815815	286898	9.44E-10	Niemann-Pick disease
Itga7	7	2230364	2231454	81008	4.37E-10	Integrin
Grina	7	114277354	114278461	266668	1.21E-10	Glutamate receptor
**L3mbtl2**	**7**	**120014365**	**120015355**	**300320**	**1.63E-11**	**L(3)mbt-like 2 (Drosophila)**
Carm1	8	20649612	20650307	363026	1.78E-19	Coactivator-associated arginine methyltransferase 1
**Rgl3**	**8**	**21092037**	**21092637**	**300444**	**1.10E-07**	**Ral guanine nucleotide dissociation stimulator-like 3**
**Pigb**	**8**	**77794848**	**77795448**	**315807**	**3.48E-24**	**Phosphatidylinositol glycan anchor biosynthesis**
Klhdc3	9	10052217	10053207	363192	5.74E-10	Kelch domain containing 3
**Sema4c**	**9**	**35501103**	**35501703**	**301346**	**1.13E-06**	**Sema domain**
**RGD1306625**	**10**	**17693855**	**17694455**	**360508**	**1.91E-07**	**Loc360508**
**Leap2**	**10**	**38883617**	**38884542**	**497901**	**7.72E-12**	**Liver-expressed antimicrobial peptide 2**
Efnb3	10	56379485	56380383	360546	1.23E-10	Ephrin B3
**Fam134c**	**10**	**90138165**	**90138765**	**360632**	**2.49E-08**	**Family with sequence similarity 134**
Sgsm1	12	44329558	44330263	288743	3.50E-21	Small G protein signaling modulator 1
**Slc4a4**	**14**	**20738777**	**20739572**	**84484**	**1.11E-18**	**Solute carrier family 4 (anion exchanger)**
**Selm**	**14**	**84159679**	**84160888**	**498398**	**3.90E-15**	**Selenoprotein M**
Mtmr7	16	54982063	54982663	306490	4.13E-13	Myotubularin related protein 7
Hist1h2bn	17	50352153	50352753	291157	9.04E-17	Histone cluster 1
**Snrpd1**	**18**	**2001020**	**2001620**	**291794**	**2.44E-19**	**Small nuclear ribonucleoprotein D1**
**Cklf**	**19**	**663953**	**664553**	**245978**	**2.04E-08**	**Chemokine-like factor**
Siah1a	19	21716960	21717780	140941	7.78E-12	Seven in absentia 1A
**Tubb3**	**19**	**53742902**	**53743830**	**246118**	**9.53E-10**	**Tubulin**
**RT1-CE5**	**20**	**3513158**	**3513758**	**309607**	**4.95E-11**	**RT1 class I**
**Vps52**	**20**	**5083014**	**5084511**	**25218**	**4.19E-09**	**Vacuolar protein sorting 52 homolog (S. Cerevisiae)**
**AA926063**	**20**	**5139498**	**5140597**	**294284**	**6.22E-30**	**Aa926063gene**
**Ptges3l1**	**X**	**63914478**	**63915574**	**367808**	**1.88E-09**	**Prostaglandin E synthase 3-like 1**

Epimutations found in F3-generation sperm after exposure of F0 generation gestating females to DDT, obtained by intersection of the results of three MeDIP-Chip comparative hybridizations. The bolded genes were found to be unique DMR associated only with DDT induced DMR.
