# Supplementary material for: Ancestral dichlorodiphenyltrichloroethane (DDT) exposure promotes epigenetic transgenerational inheritance of obesity
Source: BMC Med. 2013 Oct 23;11:228. doi: 10.1186/1741-7015-11-228 (PMC3853586; doi:10.1186/1741-7015-11-228)
Supplement: Additional file 9: Table S7 — Dichlorodiphenyltrichloroethane (DDT) induced F3 generation sperm differential DNA methylation regions (DMR) (average). Average found in F3-generation rat sperm after exposure of F0 generation to DDT, obtained averaging the results of three comparative hybridizations. [file 1741-7015-11-228-S9.pdf]

Supplemental Table S7.

DDT induced F3 generation sperm differential DNA methylation regions (DMR) (Average)

Gene Symbol	Chr	Start	End	Gene ID	Min P Value	Gene Title
RGD1565772	1	67650634	67651234	308341	2.26E-06	Similar to hypothetical protein A430110N23
Kptn	1	76460071	76460966	308107	1.88E-07	Kaptin (actin binding protein)
Dmpk	1	78450672	78451272	308405	8.74E-07	Dystrophia myotonica-protein kinase
Plaur	1	79705421	79706021	50692	6.94E-06	Plasminogen activator
Znf575	1	79870598	79872243	308430	2.34E-17	Zinc finger protein 575
Ethe1	1	79875744	79876439	292710	4.22E-07	Ethylmalonic encephalopathy 1
Cyp2t1	1	82230254	82230854	171380	3.49E-06	Cytochrome P450
Fxyd5	1	86084554	86085239	60338	2.19E-06	FXYD domain-containing ion transport regulator 5
Klk8	1	94226280	94226880	308565	2.02E-07	Kallikrein related-peptidase 8
Tbc1d17	1	95321318	95322308	292886	3.35E-06	TBC1 domain family
Ap2a1	1	95403348	95403948	308578	9.46E-06	Adaptor-related protein complex 2
Rpl13a	1	95603462	95604158	317646	6.12E-06	Ribosomal protein L13A
Trpm4	1	95802352	95803340	171143	1.35E-11	Transient receptor potential cation channel
Hrc	1	95809421	95810100	292905	2.92E-07	Histidine rich calcium binding protein
Ruvbl2	1	95907092	95907692	292907	1.40E-06	Ruvb-like 2 (E. Coli)
Sult2b1	1	96264519	96265219	292915	6.62E-06	Sulfotransferase family
Fah	1	140875586	140876369	29383	8.39E-06	Fumarylacetoacetate hydrolase
Tsku	1	155621758	155622558	308843	4.07E-21	Tsukushin
Serpinh1	1	156673272	156674075	29345	1.54E-06	Serine (or cysteine) peptidase inhibitor
Pde2a	1	158951586	158952186	81743	1.24E-07	Phosphodiesterase 2A
Aqp8	1	182331155	182331845	29172	2.72E-06	Aquaporin 8
RGD1305592	1	186199558	186200158	293500	3.58E-07	Similar to RIKEN cdna 2900092E17
Bet1l	1	201012554	201013527	54400	3.14E-06	Blocked early in transport 1 homolog
Pnpla2	1	201642430	201643110	361676	9.81E-06	Patatin-like phospholipase domain containing 2
Actn3	1	207491443	207492749	171009	1.95E-06	Actinin alpha 3
Prdx5	1	209586342	209587138	113898	1.13E-12	Peroxiredoxin 5
Loxl4	1	248268364	248269065	309380	1.11E-07	Lysyl oxidase-like 4
Sfxn3	1	250111583	250112503	65042	8.87E-09	Sideroflexin 3
Ccno	2	44376971	44377571	499528	7.78E-08	Cyclin O
Prkaa1	2	54327367	54328264	65248	8.10E-09	Protein kinase
Fxr1	2	120329766	120330366	361927	5.04E-07	Fragile X mental retardation
Muc1	2	181399893	181400493	24571	1.38E-07	Mucin 1
Kcnn3	2	181715841	181716441	54263	3.15E-06	Potassium /calcium-activated channel
Mov10	2	200075591	200076191	310756	5.97E-06	Moloney leukemia virus 10
Gstm1	2	203580702	203581302	24423	6.54E-06	Glutathione S-transferase mu 1
Extl2	2	212162933	212163821	310803	1.19E-06	Exostoses (multiple)-like 2
Dkk2	2	229541108	229541708	295445	4.25E-06	Dickkopf homolog 2 (Xenopus laevis)
Cyr61	2	243826123	243826723	83476	3.92E-06	Cysteine-rich
Ndor1	3	3420722	3421402	311799	2.09E-07	NADPH dependent diflavin oxidoreductase 1
Zdhhc12	3	9099292	9099892	366014	3.16E-06	Zinc finger
Ppp6c	3	18997054	18997654	171121	2.50E-11	Protein phosphatase 6
Epc2	3	30420205	30420805	362132	8.61E-07	Enhancer of polycomb homolog 2 (Drosophila)
Wipf1	3	56002074	56002870	117538	2.32E-06	WAS/WASL interacting protein family
Lrrc55	3	68429303	68429903	311171	7.74E-06	Leucine rich repeat containing 55
Arfgap2	3	75620106	75620706	362162	5.38E-06	ADP-ribosylation factor gtpase activating protein 2

Chrm4	3	76302545	76303245	25111	7.84E-08	Cholinergic receptor
Gyltl1b	3	76768724	76769324	311202	4.26E-06	Glycosyltransferase-like 1B
Aqr	3	100034436	100035036	366163	6.11E-06	Aquarius homolog (mouse)
Meis2	3	102118424	102119024	311311	1.11E-06	Meis homeobox 2
Cdan1	3	107508165	107508765	311348	5.72E-06	Congenital dyserythropoietic anemia
Sord	3	109012700	109013590	24788	1.13E-09	Sorbitol dehydrogenase
Duox2	3	109076069	109076669	79107	1.38E-06	Dual oxidase 2
Cst3	3	137654243	137654843	25307	9.67E-06	Cystatin C
Slc32a1	3	149342639	149343525	83612	6.77E-09	Solute carrier family 32 (GABA transporter)
Spata2	3	158640784	158641479	114210	4.37E-12	Spermatogenesis associated 2
Cyp24a1	3	161553464	161554064	25279	6.85E-06	Cytochrome P450
Rps21	3	169311014	169311614	81775	5.96E-09	Ribosomal protein S21
Ppdpf	3	170326330	170326930	296470	3.28E-06	Pancreatic progenitor cell differentiation
Slc4a2	4	6116769	6117673	24780	3.89E-07	Solute carrier family 4 (anion exchanger)
Impdh1	4	56086107	56086807	362329	2.69E-06	IMP (inosine monophosphate) dehydrogenase 1
Dok1	4	117244893	117245884	312477	1.19E-09	Docking protein 1
Htra2	4	117264316	117265101	297376	2.08E-09	Htra serine peptidase 2
Pcgf1	4	117290323	117291507	312480	4.55E-07	Polycomb group ring finger 1
Abtb1	4	122936708	122937308	297432	1.38E-06	Ankyrin repeat and BTB (POZ) domain 1
Gpr27	4	134542013	134542613	65275	1.59E-10	G protein-coupled receptor 27
Ret	4	154491475	154492462	24716	1.46E-06	Ret proto-oncogene
Clstn3	4	160665062	160665757	171393	3.18E-06	Calsyntenin 3
Gapdh	4	161284964	161285858	24383	3.54E-09	Glyceraldehyde-3-phosphate dehydrogenase
Ntf3	4	162506324	162506924	81737	5.85E-06	Neurotrophin 3
Kcna1	4	163015524	163016204	24520	3.95E-07	Potassium voltage-gated channel
Csda	4	169099521	169100121	83807	2.43E-09	Cold shock domain protein A
Tuba8	4	1634530	1635405	500377	9.66E-07	Tubulin
RGD1309821	5	58926895	58927782	366360	2.56E-07	Similar to KIAA1161 protein
Sit1	5	59964375	59965190	500449	3.11E-07	Signaling threshold transmembrane adaptor 1
Nipsnap3b	5	70448849	70449561	313211	6.19E-12	Nipsnap homolog 3B (C. Elegans)
Pole3	5	79520387	79520987	298098	8.74E-08	Polymerase (DNA directed)
Kank4	5	119232056	119232656	313385	7.01E-07	KN motif and ankyrin repeat domains 4
Faah	5	136310905	136312105	29347	1.69E-11	Fatty acid amide hydrolase
Cdc20	5	138916520	138917300	64515	3.00E-06	Cell division cycle 20 homolog (S. Cerevisiae)
Hcrtr1	5	149170407	149171007	25593	3.11E-11	Hypocretin (orexin) receptor 1
Mrto4	5	158154393	158155393	298586	5.13E-09	Mrna turnover 4 homolog (S. Cerevisiae)
Efhd2	5	160776270	160776955	298609	9.13E-10	EF-hand domain family
Pik3cd	5	166762948	166763658	366508	3.83E-06	Phosphoinositide-3-kinase
Cyp1b1	6	2552849	2553544	25426	4.60E-06	Cytochrome P450
Rhoq	6	10413845	10414445	85428	1.58E-09	Ras homolog gene family
Slc30a3	6	25256014	25256614	366568	3.96E-06	Solute carrier family 30 (zinc transporter)
Dnmt3a	6	26859684	26860575	444984	1.24E-06	DNA (cytosine-5-)-methyltransferase 3 alpha
Adcy3	6	27118646	27119246	64508	8.81E-06	Adenylate cyclase 3
Pygl	6	92340804	92341404	64035	5.01E-06	Phosphorylase
Hspa2	6	99000603	99001203	60460	3.37E-06	Heat shock protein alpha 2
Rdh11	6	101962268	101962868	362757	4.62E-06	Retinol dehydrogenase 11
Npc2	6	108815006	108815815	286898	5.94E-07	Niemann-Pick disease
Batf	6	109793090	109794280	299206	3.23E-09	Basic leucine zipper transcription factor
Esrrb	6	110585981	110586661	299210	2.93E-07	Estrogen-related receptor beta

Ckb	6	136459281	136459966	24264	6.06E-06	Creatine kinase
Trmt61a	6	136460561	136461161	314462	4.72E-06	Trna methyltransferase 61 homolog A
Akt1	6	137660231	137661023	24185	1.56E-08	V-akt murine thymoma viral oncogene homolog 1
Itga7	7	2230364	2231454	81008	4.77E-09	Integrin
Pip5k1c	7	9908530	9909318	314641	3.21E-07	Phosphatidylinositol-4-phosphate 5-kinase
Hcn2	7	11503885	11504585	114244	1.30E-07	Hyperpolarization activated cyclic nucleotide-gated
Ccdc53	7	24894041	24894825	299707	2.79E-06	Coiled-coil domain containing 53
Geft	7	67143342	67144162	314904	5.01E-06	Rhoa/RAC/CDC42 exchange factor
Ddit3	7	67247684	67248284	29467	1.67E-07	DNA-damage inducible transcript 3
Eif3h	7	88154143	88154743	299899	2.87E-06	Eukaryotic translation initiation factor 3
Grina	7	114277354	114278461	266668	7.23E-12	Glutamate receptor
Fbxl6	7	114590568	114591268	362941	1.10E-06	F-box and leucine-rich repeat protein 6
L3mbtl2	7	120013411	120014965	300320	3.48E-06	L(3)mbt-like 2 (Drosophila)
Serhl2	7	121125849	121126559	500911	4.76E-08	Serine hydrolase-like 2
Tubgcp6	7	127422817	127423417	362980	5.76E-06	Tubulin
Ccdc65	7	137475638	137476238	362994	5.34E-06	Coiled-coil domain containing 65
Grasp	7	139968182	139968967	192254	7.69E-06	GRP1 (general receptor for phosphoinositides 1)-
Mfsd5	7	141033250	141033850	315329	4.03E-07	Major facilitator superfamily domain containing 5
Zfp385a	7	142249740	142250435	685474	3.74E-14	Zinc finger protein 385A
Sesn3	8	11075535	11076545	315427	6.39E-16	Sestrin 3
Carm1	8	20649707	20650307	363026	4.16E-09	Coactivator-assoc arginine methyltransferase 1
Rgl3	8	21091844	21092637	300444	1.15E-08	Ral guanine nuc dissociation stimulator-like 3
Ccdc151	8	21106911	21107511	315465	6.07E-06	Coiled-coil domain containing 151
Kcnj5	8	32104069	32104669	29713	5.00E-06	Potassium inwardly-rectifying channel
Ets1	8	32481718	32482437	24356	1.37E-07	V-ets erythroblastosis virus E26 oncogene
Kirrel3	8	34300842	34301624	315546	6.40E-11	Kin of IRRE like 3 (Drosophila)
Bcl9l	8	47449901	47450616	300673	6.65E-07	B-cell CLL/lymphoma 9-like
Usp28	8	52245410	52246190	315639	1.00E-08	Ubiquitin specific peptidase 28
Pts	8	53890354	53890954	29498	6.05E-09	6-pyruvoyl-tetrahydropterin synthase
Pstpip1	8	59693388	59694278	300732	1.08E-06	Proline-serine-threonine phosphatase-interacting
Lingo1	8	60353691	60354291	315691	1.14E-06	Leucine rich repeat and Ig domain containing 1
Rora	8	73052515	73053115	300807	3.85E-07	RAR-related orphan receptor A
Pigb	8	77794848	77795448	315807	1.39E-10	Phosphatidylinositol glycan anchor biosynthesis
Grm2	8	111848912	111849992	24415	1.10E-08	Glutamate receptor
Tmie	8	115269500	115270189	501061	1.88E-08	Transmembrane inner ear
Klhdc3	9	10052217	10053207	363192	1.68E-10	Kelch domain containing 3
Yipf3	9	10298009	10298609	301245	3.93E-07	Yip1 domain family
Sema4c	9	35501103	35501993	301346	4.49E-07	Sema domain
Dnpep	9	74594494	74595372	301529	3.24E-06	Aspartyl aminopeptidase
Itm2c	9	84611904	84612604	301575	4.73E-07	Integral membrane protein 2C
Ecel1	9	85945765	85946448	60417	3.94E-08	Endothelin converting enzyme-like 1
Lrrfip1	9	90277707	90278387	367314	1.00E-07	Leucine rich repeat (in FLII) interacting protein 1
Farp2	9	92791518	92792418	316639	4.65E-06	Ferm
Thoc6	10	12941568	12942463	79227	3.84E-09	THO complex 6 homolog (Drosophila)
Fam173a	10	15058618	15060194	287150	4.33E-12	Family with sequence similarity 173
RGD1306625	10	17693855	17694455	360508	1.65E-07	Loc360508
Adra1b	10	28946047	28946647	24173	1.74E-06	Adrenergic
Phf15	10	37379987	37380587	303113	3.96E-06	PHD finger protein 15
Leap2	10	38883617	38884452	497901	2.79E-11	Liver-expressed antimicrobial peptide 2

Kdm6b	10	56203971	56205090	363630	6.09E-07	Lysine (K)-specific demethylase 6B
Efnb3	10	56379485	56380383	360546	7.33E-13	Ephrin B3
Bcl6b	10	57068793	57069487	360551	1.59E-06	B-cell CLL/lymphoma 6
RGD1308134	10	57077341	57078127	287452	5.66E-07	Similar to RIKEN cdna 1110020A23
Pfn1	10	57534113	57534810	64303	1.35E-06	Profilin 1
Olr1512	10	61444527	61445127	287511	8.87E-07	Olfactory receptor 1512
Flot2	10	64080542	64081256	83764	4.44E-06	Flotillin 2
Ksr1	10	65321023	65321623	360573	8.45E-06	Kinase suppressor of ras 1
Abr	10	67617843	67618626	287537	1.21E-06	Active BCR-related gene
Tbx2	10	74084425	74089738	303398	2.81E-09	T-box 2
Hoxb4	10	85050638	85051430	497988	9.82E-06	Homeo box B4
Psmb3	10	86452640	86453240	29676	7.60E-06	Proteasome (prosome
Nr1d1	10	87547384	87548384	252917	4.34E-07	Nuclear receptor subfamily 1
Krt24	10	88188780	88189657	287675	2.08E-10	Keratin 24
Kat2a	10	89651442	89652042	303539	9.32E-06	K(lysine) acetyltransferase 2A
Fam134c	10	90138165	90138765	360632	1.54E-08	Family with sequence similarity 134
Brca1	10	90572480	90573080	497672	7.94E-08	Breast cancer 1
Smurf2	10	96269129	96269729	303614	5.23E-06	SMAD specific E3 ubiquitin protein ligase 2
Nat9	10	105260764	105261650	303669	3.86E-07	N-acetyltransferase 9 (GCN5-related
Ush1g	10	105399847	105400642	287819	1.54E-06	Usher syndrome 1G homolog (human)
Caskin2	10	105914751	105915351	303678	1.79E-07	Cask-interacting protein 2
Sectm1a	10	110295386	110296167	287885	1.52E-07	Secreted and transmembrane 1A
RGD1310778	11	30708849	30709564	288272	6.59E-08	Similar to Putative protein c21orf45
Setd4	11	33788133	33788830	245975	2.46E-07	SET domain containing 4
Pvrl3	11	55843915	55844515	288124	1.62E-06	Poliovirus receptor-related 3
Abcf3	11	82581404	82582483	287982	3.00E-09	ATP-binding cassette
Thap7	11	85474161	85474844	287944	6.27E-06	THAP domain containing 7
Asmt	12	16816154	16816847	246281	6.90E-10	Acetylserotonin O-methyltransferase
Il3ra	12	16829845	16830445	246144	3.86E-09	Interleukin 3 receptor
Mcm7	12	17614097	17614983	288532	3.69E-06	Minichromosome component 7
Vgf	12	20901963	20902948	29461	5.74E-06	VGF nerve growth factor inducible
Gtf2ird1	12	23319136	23319820	246770	5.42E-06	GTF2I repeat domain containing 1
Slc24a6	12	37250073	37250673	498185	4.99E-07	Solute carrier family 24 (sodium/potassium/calcium
Gltp	12	43194232	43194832	288707	8.37E-06	Glycolipid transfer protein
Sgsm1	12	44329558	44330263	288743	8.30E-13	Small G protein signaling modulator 1
Tomm40b	13	87111588	87112502	304971	7.32E-08	Translocase of outer mitochondrial membrane 40
Ppox	13	87173617	87174403	289219	7.18E-08	Protoporphyrinogen oxidase
Fgfrl1	14	1564558	1565243	360903	1.61E-06	Fibroblast growth factor receptor-like 1
Slc4a4	14	20738777	20739572	84484	4.05E-07	Solute carrier family 4 (anion exchanger)
Gabra4	14	39047535	39048135	140675	5.17E-06	Gamma-aminobutyric acid (GABA) A receptor
Qdpr	14	70741467	70742543	64192	3.36E-07	Quinoid dihydropteridine reductase
Selm	14	84159679	84160888	498398	4.82E-15	Selenoprotein M
Gatsl3	14	84841158	84842039	360969	1.75E-07	GATS protein-like 3
Nefh	14	85605152	85605752	24587	1.35E-06	Neurofilament
Zrsr1	14	103656314	103656914	498425	3.86E-09	Zinc finger (CCCH type)
Ndrg2	15	27346660	27347460	171114	1.88E-06	N-myc downstream regulated gene 2
Map1s	16	19026425	19027110	290640	3.28E-06	Microtubule-associated protein 1S
Tssk6	16	20011390	20011990	290670	1.86E-07	Testis-specific serine kinase 6
Lpar2	16	20085235	20086235	498609	1.82E-08	Lysophosphatidic acid receptor 2

Mtmr7	16	54982063	54982663	306490	3.05E-07	Myotubularin related protein 7
Cldn23	16	60109078	60109863	290789	3.62E-06	Claudin 23
Plat	16	73735602	73736590	25692	4.95E-06	Plasminogen activator
Higd2a	17	16084231	16084831	290999	8.04E-06	HIG1 hypoxia inducible domain family
Bicd2	17	21261287	21261965	306809	1.85E-06	Bicaudal D homolog 2 (Drosophila)
Edn1	17	28311655	28312632	24323	7.28E-07	Endothelin 1
Hist1h2bn	17	50352153	50352753	291157	8.37E-09	Histone cluster 1
Snrpd1	18	2001020	2001620	291794	8.10E-16	Small nuclear ribonucleoprotein D1
Psd2	18	28595608	28596208	307500	6.56E-06	Pleckstrin and Sec7 domain containing 2
Zmat2	18	29493454	29494054	679898	7.74E-07	Zinc finger
Hdac3	18	30875398	30876374	84578	1.14E-06	Histone deacetylase 3
Mc5r	18	64679777	64680673	25726	3.36E-08	Melanocortin 5 receptor
Cklf	19	663953	664553	245978	1.15E-06	Chemokine-like factor
Gpr56	19	10474868	10475468	260326	8.48E-06	G protein-coupled receptor 56
Mt1a	19	11262202	11262802	24567	1.41E-07	Metallothionein 1a
Mt2A	19	11268160	11268760	689415	5.00E-07	Metallothionein 2A
Siah1a	19	21716960	21717780	140941	4.56E-07	Seven in absentia 1A
Tppp3	19	35287184	35287884	291966	2.15E-06	Tubulin polymerization-promoting protein family
Marveld3	19	39829993	39830671	498950	5.77E-06	MARVEL domain containing 3
Plcg2	19	47611532	47612132	29337	3.49E-09	Phospholipase C
Tubb3	19	53742902	53743830	246118	4.83E-11	Tubulin
Ppp1r10	20	2984169	2984883	65045	6.21E-08	Protein phosphatase 1
RT1-CE5	20	3513158	3513758	309607	6.05E-13	RT1 class I
RT1-CE4	20	3538680	3539280	414783	8.17E-07	RT1 class I
Agpat1	20	4246520	4248196	406165	3.69E-10	1-acylglycerol-3-phosphate O-acyltransferase 1
Pbx2	20	4259521	4260219	406164	1.36E-09	Pre-B-cell leukemia homeobox 2
Gpsm3	20	4264786	4265476	406163	1.28E-06	G-protein signaling modulator 3 (AGS3-like
Psmb8	20	4789138	4790128	24968	2.68E-10	Proteasome (prosome
Vps52	20	5083014	5084511	25218	1.31E-09	Vacuolar protein sorting 52 homolog
Wdr46	20	5097321	5098324	309628	1.03E-06	WD repeat domain 46
AA926063	20	5139498	5140393	294284	5.37E-14	Aa926063gene
Kifc1	20	5147332	5147932	294286	1.50E-08	Kinesin family member C1
RGD1564450	20	5774457	5775360	294291	7.14E-06	Rgd1564450
Glp1r	20	9224920	9225807	25051	1.63E-06	Glucagon-like peptide 1 receptor
RGD1303003	20	10850846	10851446	294326	2.07E-06	Homolog of zebrafish ES1
Derl3	20	13164828	13165506	690315	8.28E-06	Der1-like domain family
RT1-CE16	20	39700	40300	414819	3.19E-06	RT1 class I
RT1-CE11	20	131360	131960	414791	3.76E-06	RT1 class I
Ptges3l1	X	63914885	63915485	367808	1.12E-06	Prostaglandin E synthase 3-like 1

Average found in F3-generation rat sperm after exposure of F0 generation to DDT, obtained averaging the results of three comparative hybridizations.
